# Temporal depressive symptom networks in older adults during the COVID-19 pandemic

**DOI:** 10.1016/j.xjmad.2025.100156

**Published:** 2025-11-07

**Authors:** Michael Odenthal, Pascal Schlechter, Christoph Benke, Christiane A. Melzig

**Affiliations:** a University of Marburg, Marburg, Germany; b University of Münster, Münster, Germany

**Keywords:** COVID-19 pandemic, Network analysis, CLPN, Depression, Mental health, Somatic symptoms, The English Longitudinal Study of Ageing

## Abstract

**Background:**

Older adults show high rates of depression with an increase during the COVID-19 pandemic. Social isolation and lifestyle disruptions contributed to loneliness, symptoms of depression, and reduced activity. For interventions, it is crucial to understand the development of symptoms of depression networks and changes over time under stressors such as pandemics. Yet, no COVID-19 longitudinal depression network study in older people exists. We therefore aimed to investigate changes in relationships between symptoms of depression in older adults during compared to the pre-COVID-19 period.

**Methods:**

We conducted cross-lagged panel network analyses in four waves from the English Longitudinal Study of Ageing (ELSA), a representative dataset of the UK population over 50 years (initial *N* = 7223). We examined networks (1) between two pre-pandemic points, (2) from the second pre-pandemic to the first pandemic wave as maximum stressor, and (3) between two pandemic waves as repeated stressor.

**Results:**

While the pre- and pandemic networks showed consistent overall structures, some differences emerged. In the pre-pandemic network, *feeling lonely* and *could not get going* were the only nodes with positive out-expected influence, suggesting a central activating role. During the pandemic, additional symptoms developed stronger outgoing connections, suggesting a shift toward a broader set of symptoms driving network dynamics under prolonged stress.

**Discussion:**

Loneliness appears to play a key role in symptom development under acute and repeated stress. Findings suggest that dynamics in symptoms of depression networks in older adults are relatively stable, even during acute and repeated stress during the COVID-19 pandemic.

## Introduction

Depression is highly prevalent among people over 50 in Europe and North America, with meta-analytical prevalence estimates ranging from 13.3 % [1] to 16.5 % [2]. In older populations, depression frequently manifests through somatic symptoms and overlapping medical conditions [3]. Importantly, early symptoms of depression in older adults may differ from typical diagnostic criteria, with somatic symptoms such as lack of energy and decreased drive often being prevalent [4]. Considerable efforts have been made to identify psychosocial factors that may contribute to the development and maintenance of symptoms of depression in later life. For example, loneliness has been shown to significantly contribute to the development and maintenance of symptoms of depression in older populations [5], [6], [7], [8], [9], [10]. More specifically, a major factor that has received growing attention is stress: acute and chronic stress have been demonstrated to play a central role in the onset and maintenance of symptoms of depression, particularly in vulnerable populations [11]. Older people often experience major life transitions, such as role changes, retirement, or the death of loved ones [12], which can affect symptoms of depression. One of the most significant recent stressors was the COVID-19 pandemic which caused widespread disruptions in social, physical, and psychological domains. Since its outbreak, rates of depression have increased 2–8 times (for a meta-analysis, see [13]). During COVID-19, government policies have led to declines in social networks, while COVID-19 increased illness and death, especially among older people [14]. The pandemic introduced unprecedented and multifaceted stressors, particularly for older people who may be vulnerable due to isolation, health concerns, and changes in daily routines [15], [16], [17].

While many studies have investigated symptoms of depression in the general or in younger populations during the pandemic [18], [19], [20], [21], [22], older adults remain underexamined although they face specific stressors such as chronic illness, social loss, and somatic vulnerability. To investigate changes in depression during the COVID-19 pandemic in older people, current research [15], [23], [10] mainly examined average differences and prevalence estimates from a common cause perspective (i.e., treating symptoms as interchangeable indicators of depression). Meta-analytic findings indicate that mental health in older adults declined slightly in the early pandemic phase, with signs of later recovery comparable to those of younger adults [24]. While such approaches provide prevalence data, they do not capture the dynamic interplay between symptoms of depression. This approach is therefore not well-suited to adequately examine complex relationships among symptoms of depression over time, which is important for developing more targeted prevention and intervention strategies [25], [26].

Given a potential higher vulnerability of older adults to symptoms of depression under stress, it is essential to investigate how interactions among symptoms of depression evolve during high-stress periods such as the COVID-19 pandemic. The network approach can offer nuanced insights into complex and dynamic relationships between individual symptoms by modeling psychological symptoms as interrelated nodes that can activate or reinforce themselves and one another [25], [26]. According to the network approach to psychopathology [27], [25], psychological symptoms, i.e., nodes, are interconnected to varying degrees (interconnections are referred to as edges). Central core symptoms of depression have a pronounced effect on other mental health related symptoms in a network. Finding network key symptoms could thus be clinically important as they can potentially activate the entire network, increasing overall symptom severity [28]. By exploring these dynamics and identifying central core symptoms of depression within depressive symptom networks, especially by examining how these networks shift in response to external perturbations as acute or chronic stress, we aimed to gain critical insights into context-sensitive mechanisms of symptom emergence and maintenance. Beyond nosological frameworks focusing on descriptive diagnostic criteria, these networks reflect interrelations among symptoms typically associated with depression. Such insights may inform targeted interventions addressing specific pathogenic pathways in older adults.

In this context, longitudinal analyses are necessary to analyze temporal interactions of acute and repeated stressors. As acute stressors like the pandemic outbreak may perturbate established symptom dynamics [29], these stressors can exacerbate emotional distress over time, leading to a vicious circle of deteriorating mental health [30]. Repeated stressors like ongoing restrictions and prolonged disruptions in daily routines may reinforce or shift symptom associations over time. This may contribute to an increased vulernability to symptoms of depression among older adults, if symptoms of depression networks get more easily activated [31]. So far, mainly cross-sectional network studies have examined symptoms of depression in older populations during the pandemic with inconsistent findings. These studies revealed either high centralities of *nervousness* and *excessive worry* [32], [33] or of *sad mood, guilt, motor, lack of energy* as central symptoms [34]. Longitudinal data in older populations have been limited to the pre-pandemic period yet. In the most comprehensive temporal network analysis in older people to date, Schlechter et al. [9] analyzed symptoms of depression in 11,391 adults aged 50 and above from the English Longitudinal Study of Ageing (ELSA) over 16 years from 2002 to 2019 using Cross-Lagged Panel Network Models (CLPN). In this study, the Center for Epidemiologic Studies Depression Scale – 8 (CES-D-8) was used, which assesses the presence of eight depressive symptoms over the past week, including both affective (e.g., *feeling depressed, feeling sad*) and somatic symptoms (e.g., *everything was an effort, could not get going, restless sleep*). Findings revealed consistent networks over time with *everything was an effort* and *could not get going* showing the strongest reciprocal associations. These symptoms and *feeling lonely* had emerged as core symptoms, as they lead to the activation of many other network symptoms and were also activated by other mental health related symptoms. In contrast, the DSM-V [35] hallmark symptom *feeling depressed* was influenced by other symptoms of depression but did not activate new symptoms. *Restless sleep* primarily preceded other symptoms of depression, suggesting that certain symptoms of depression may emerge earlier than others in older adults. This points to potential somatic pathways towards depression in older people and these early-emerging symptoms may serve as targets for screening or early intervention, especially in individuals at higher risk for depression.

In addition to cross-sectional network data in older adults during the COVID-19 pandemic and pre-pandemic longitudinal network data, a first study [36] has examined changes in dynamic symptom networks in the general population under pandemic conditions. For example, in a previous study in the general population in the United Kingdom (mean age 49.85–54.82 years), we demonstrated that *feeling lonely* and *feeling worthless* had a strong influence on other symptoms of depression, thus constituting central precursor symptoms. In contrast, *feeling depressed* and *not overcoming difficulties* had many incoming connections, thus constituting an end product of symptom cascades [36]. These associations emerged in a period of maximum stress (pre-pandemic to pandemic onset) and repeated stress (over pandemic peak). While this study provided important insights into symptom dynamics during the pandemic in the general population, it remains unclear how such dynamics unfold specifically in older adults, as they may be particularly affected by COVID-19-related stressors due to pre-existing health conditions, social isolation and major life transitions (see above). Therefore, the present study aimed to examine the dynamics of depressive symptom networks in older people, with a particular focus on their potential change in response to external perturbations during the COVID-19 pandemic.

Importantly, ELSA has continued into the COVID-19 pandemic, but data have not been analyzed from a network perspective. The current study therefore uses longitudinal ELSA data to examine whether symptom networks changed from the pre-pandemic temporal network (network 1) to a phase of acute stress (pre-pandemic to pandemic outbreak, network 2), to a period of repeated stress (during the pandemic, network 3). Accordingly, we examine whether stressors not only exacerbate existing symptoms but may also shift symptom networks’ structure by enhancing central symptoms or leading to new associations between symptoms. Consistent symptom associations across phases would suggest robust network structures, supporting interventions targeting core symptoms such as *everything was an effort* or *feeling lonely*. In contrast, the emergence of new associations would highlight the unique mental health impact of pandemic-related stress in older adults.

## Methods

### Participants and study design

We conducted a secondary data analysis based on the core ELSA sample, a study that examines the interrelationships between health, social, wellbeing and economic circumstances in the English population aged 50 and older [37]. ELSA collected data from private households in England with participants drawn from the Health Survey for England (HSE). This study offers a representative sample of the population aged 50 years and older, beginning in 2002/2003. A multistage stratified probability sampling method was employed, with new participants being added over time to ensure representativeness. The core sample comprises 11,391 adults born on or before 29 February 1952 who initially participated in the HSE. Participants were eligible for all waves, but not all participants provided data in each wave. In order to obtain networks from the same people over time, the present analyses focus on the core sample members with repeated-measures data. Table 1 depicts the demographic characteristics for each ELSA wave. As we examined the temporal dynamics of mental health over the beginning of the COVID-19 pandemic and compared this with mental health during the last two waves before the pandemic started, we focused on wave 8 collected from May 2016 to June 2017, wave 9 collected from June 2018 to July 2019, COVID-19 wave 1 collected from June to July 2020, and COVID-19 wave 2 collected from November to December 2020. The COVID-19 waves 1 (June–July 2020) and 2 (November–December 2020) represent a subsample of the core sample (aged ≥ 50, HSE origin, Wave 1 participants). Ethical approval was granted by the National Research Ethics Service (MREC/01/2/91). Participants provided informed consent. The data are openly available via the UK Data Service. Our secondary data analysis was not preregistered.

### Questionnaire measures

The CES-D-8 (Center for Epidemiologic Studies Depression Scale - 8) is a brief self-report instrument for assessing symptoms of depression in the general population, administered in all eleven ELSA waves [38]. The CES-D-8 comprises eight items that measure the occurrence of symptoms of depression in the past week in a dichotomous response format (yes/no). Respondents indicate whether they have experienced each symptom in the past week with total scores ranging from 0 (no symptoms) to 8 (all symptoms present). It covers depressive affect (*enjoyed life, felt depressed, happy, lonely,* and *felt sad*) and somatic complaints (*everything was an effort, restless sleep,* and *could not get going*). The items *happiness* and *enjoying life* are positively worded. Unlike the DSM-5 criteria for major depressive disorder [35], the CES-D-8 captures symptom presence without establishing a formal diagnosis. Therefore, the CES-D-8 provides information on the presence of typical depressive symptoms in the population, allowing for the examination of symptom dynamics and interrelations rather than diagnostic classification. Despite its concise form, the CES-D-8 retains robust psychometric properties comparable to the original CES-D-20 scale, including high internal consistency and validity [39], as well as longitudinal measurement invariance across time in this population [40]. Specifically, the sensitivity and specificity of the CES-D were 56.2 %–70.2 % and 84.7 %–94.0 %, respectively [41].

### Missingness analysis and data imputation

During the eighth wave, 7223 participants attended the study, during the ninth wave 7289, during the first COVID-19 wave 5825, and during the second COVID-19 wave 5594. Sample characteristics of the participants who dropped out of the study have been described elsewhere [9]. Missingness was not completely at random. Participants who dropped out were more likely non-white (compared to white), older, unmarried individuals (compared to married), had a lower educational level, and a higher severity of depression. This means that the likelihood of missing data is related to these observed variables. Therefore, the missing data can be categorized as missing at random (MAR), since the missingness depends on observed variables but not on unobserved ones. Hence, we conducted data imputation with a single imputed dataset [42], [43]. To include all cases in all networks, we imputed data on the CES-D-8 items for all waves. Imputation analysis was based on wave 8 data (i.e., the first wave of our analyses, *N* = 7223). Demographics thus resemble those of wave 8 (see Table 1). The MICE package in R was used [44]. In our imputation models, we incorporated the variables associated with missingness (i.e., ethnicity and sex) as auxiliary variables. This way, we aimed to reduce bias in the CES-D-8 items associated with missing data. We conducted data analyses with the imputed data. Additionally, we performed a sensitivity analysis using full cases only (see Supplemental Figure s16 and s17). The results remained mainly the same compared to the imputed data set, increasing confidence in our results and their interpretation.

**Table 1:** Number and percentage of participants endorsing the dichotomous CES-D-8 items across waves, sum scores, internal consistencies, and item-level skewness and kurtosis without imputation. The items *were happy* and *enjoyed life* were reverse-coded and included in the model, leading to negative associations.

Assessment year	05/2016–06/2017	06/2018–07/2019	06/2020–07/2020	11/2020–12/2020
1 …you felt depressed	*n* = 837 (12.13 %)	*n* = 797 (11.45 %)	*n* = 1019 (17.52 %)	*n* = 1076 (19.26 %)
2 …you felt everything you did was an effort	*n* = 1373 (19.90 %)	*n* = 1344 (19.30 %)	*n* = 1418 (24.38 %)	*n* = 1437 (25.73 %)
3 …your sleep was restless	*n* = 2516 (36.46 %)	*n* = 3006 (43.17 %)	*n* = 2596 (44.67 %)	*n* = 2663 (47.71 %)
4 …you were happy	*n* = 6272 (91.94 %)	*n* = 6306 (90.83 %)	*n* = 4865 (84.04 %)	*n* = 4520 (81.27 %)
5 …you felt lonely	*n* = 840 (12.17 %)	*n* = 774 (11.12 %)	*n* = 1064 (18.31 %)	*n* = 1050 (18.82 %)
6 …you enjoyed life	*n* = 6305 (91.46 %)	*n* = 6333 (91.19 %)	*n* = 4693 (81.17 %)	*n* = 4356 (78.37 %)
7 …you felt sad	*n* = 1350 (19.57 %)	*n* = 1261 (18.13 %)	*n* = 331 (24.02 %)	*n* = 1683 (30.20 %)
8 …you could not get going	*n* = 1360 (19.71 %)	*n* = 1330 (19.12 %)	*n* = 1612 (27.80 %)	*n* = 1711 (30.69 %)
Cronbach’s alpha, full scale (α)	0.78	0.78	0.82	0.83
Depressed affect (α)	0.76	0.77	0.80	0.81
Somatic complaints (α)	0.45	0.45	0.52	0.55
**Skewness/Kurtosis**	**Wave 8 (*****N*****= 7223)**	**Wave 9 (*****N*****= 7289)**	**COVID wave 1 (*****N*****= 5825)**	**COVID wave 2 (*****N*****= 5594)**
1 …you felt depressed	2.32/3.38	2.42/3.86	1.71/0.92	1.56/0.43
2 …you felt everything you did was an effort	1.51/0.27	1.56/0.42	1.19/−0.58	1.11/−0.77
3 …your sleep was restless	0.56/−1.68	0.28/−1.92	0.21/−1.95	0.09/−1.99
4 …you were happy	−2.87/6.26	2.83/6.00	1.86/1.45	1.60/0.57
5 …you felt lonely	2.31/3.35	2.47/4.12	1.64/0.68	1.59/0.54
6 …you enjoyed life	−2.97/6.80	2.91/6.44	1.59/0.54	1.38/−0.10
7 …you felt sad	1.53/0.35	1.65/0.74	1.21/−0.52	0.86/−1.26
8 …you could not get going	1.52/0.32	1.57/0.47	0.99/−1.02	0.84/−1.30
Omega total, full scale (ω_t_)	0.84	0.84	0.86	0.87
Depressed affect (ω_t_)	0.81	0.81	0.83	0.84
Somatic complaints (ω_t_)	0.48	0.5	0.55	0.57

### Data analysis

All data analyses were performed in RStudio 4.3.2 [45]. The R code to reproduce the current results is openly available on the OSF (https://osf.io/h4e39/overview?view_only=8b4cbfafcdaa4580a0455083c62e88c3). Cross-lagged panel network models (CLPN) were used to analyze the unfolding networks over time [43]. We analyzed three longitudinal networks: (1) pre-COVID wave 8 to wave 9, (2) pre-COVID wave 9 to COVID wave 1, (3) COVID wave 1 to COVID wave 2.

In a first analysis step, we calculated the regression coefficients for the models. Given the binary responses, logistic regression models were employed [46]. This included the calculation of autoregressive paths, which represent the prediction of a symptom at one timepoint by itself at the previous timepoint, while adjusting for all other symptoms. We then computed cross-lagged pathways, which represent the prediction of one symptom by another, adjusting for the autoregressive effects and all other symptoms. Following the binary format of our measures, we transformed the coefficients of the logistic regressions (i.e., edge weights) from log odds to odds ratios (OR). Hence, edge weights > 1 can be interpreted as positive connections, edge weights < 1 as negative connections, and edge weights = 1 as having no connection. To estimate the regression coefficients, we applied the least absolute shrinkage and selection operator (LASSO), which applies a penalization to avoid estimating spurious edges [47], [48]. We calculated the directional CLPNs with the glmnet package [49], [43]. The qgraph package [50] was used to visualize the networks using an average layout for the three different networks over time.

In order to quantify the centrality of symptoms in our directed CLPNs, we analyzed two centrality indices: in- and out-expected-influence. Out-expected influence is calculated as the sum of all outgoing symptom associations that a given item has with all other items in the subsequent wave (the degree to which a symptom predicts other symptoms). In-expected-influence quantifies the incoming symptom associations of an item with all other items (the degree to which a symptom is predicted by other symptoms). The edge weight difference test and the centrality difference test were employed to detect significant differences in the edge and centrality indices, respectively [51].

We estimated the accuracy of the edge weights by calculating 95 % confidence intervals (CIs) around each edge weight using nonparametric bootstrapping with 1000 iterations. To evaluate the reliability of our findings, we employed case-drop bootstrapping (correlation of centrality indices from the entire sample with centrality index estimates on subsets of the sample) using the *bootnet* package with 1000 iterations [47]. Furthermore, the edge weight difference test and the centrality difference test were employed to examine whether specific edges among symptoms or centrality indices of symptoms exhibited notable differences compared to other edges or centrality estimates of a symptom. The first test quantified the relative importance of specific cross-lagged symptom associations (i.e., edges) in comparison to other cross-lagged symptom associations. The second test quantified the relative importance (i.e., centrality) of specific symptoms within the networks, in comparison to other symptoms.

## Results

### Descriptive statistics

Descriptive statistics of participants endorsing the dichotomous CES-D-8 items across waves, internal consistencies, item-level skewness and kurtosis can be found in Table 1. The symptom *restless sleep* had the highest endorsement over all four waves, followed by *everything was an effort* with increasing endorsement. Endorsement of *could not get going* increased over time with the second highest endorsement during the second COVID-19 timepoint. In addition, *feeling sad* increased in endorsement and finally received the third highest endorsement at the last timepoint (in consideration of reverse coding of *were happy* and *enjoying life;* see Table 1). *Were happy* and *enjoying life* decreased significantly over time while *feeling depressed* increased significantly (for mean levels and *t*-tests see Table 2).

**Table 2:** Table showing t-values and p-values from paired samples *t*-tests on relevant variables over time. *Mean range* indicates the percentage of participants who endorsed each CES-D-8 item at each wave.

	**Network**		
**Edges**	**First to second pre-COVID**	**Second pre-COVID to first COVID**	**First COVID to second COVID**
1st strongest	everything was an effort → could not get going	were happy → enjoy life	enjoy life → were happy
2nd strongest	were happy → enjoy life	feeling lonely → feeling depressed	everything was an effort → could not get going
3rd strongest	everything was an effort → feeling depressed	everything was an effort → could not get going	could not get going → everything was an effort
4th strongest	could not get going → everything was an effort	could not get going → feeling depressed	feeling depressed → feeling lonely
5th strongest	feeling lonely → feeling depressed	Feeling depressed → sad	feeling lonely → sad

### Accuracy and stability

According to our bootstrapping results, the networks showed good accuracy, as edge weights exhibited small to moderate bootstrapped CIs (see Figures s1-s3). Furthermore, our networks exhibited high stability of the centrality measures, as shown in the bootstrapping results by case-drop procedure (see Figures s4-s6).

### Network comparison

The edge lists of all networks are presented in Table s1 in the supplemental materials. The number of non-zero edges was consistent across networks [range: 51 (second pre-COVID to first COVID network) – 62 (first pre-COVID to second pre-COVID network)]. The correlations of the edge lists between the networks were high (range: *r* = 0.69 – *r* = 0.81). The overall correlations of the out-expected-influence (*r* = 0.61 – 0.62) and the overall correlations of the in-expected-influence (*r* = 0.61 – 0.98) centrality indices between networks were high. Table 3 depicts the five strongest edges for all three networks. The strongest edge connection over time was *everything was an effort* → *could not get going* (range: OR: 2.08 – 2.57; strongest in network 1, third strongest in network 2 and second strongest in network 3). The edge connection *were happy* → *enjoy life* (range: OR: 2.10 – 2.18) emerged as second strongest in network 1, strongest in network 2, and in reverse as *enjoy life* → *were happy* as strongest in network 3, while the edge connection *feeling lonely* → *feeling depressed* increased in strength from pre-COVID to the first COVID wave (second network; range: OR: 1.93 – 2.09), while it decreased in the third network compared to the pre-pandemic network (OR: 1.77).

**Table 3:** Table depicting the 5 strongest edges for all three networks.

**Variable**	**Mean range**	**Wave 8 – Wave 9**	**Wave 9 – Covid wave 1**	**COVID wave 1 – wave 2**
*Were happy*	91.94–81.27	*t* = −0.12236, *p* = 0.9026	*t* = 13.797, *p* < 0.001	*t* 5.0352, *p* < 0.001
*Enjoying life*	91.46–78.37	*t* = 1.6954, *p* = 0.090	*t* = 19.182, *p* < 0.001	*t* = 4.6576, *p* < 0.001
*Feeling depressed*	12.13–19.26	*t* = 0.51884, *p* = 0.604	*t* = −13.419, *p* < 0.001	*t* = −3.318, *p* < 0.001

**Fig. 1:**
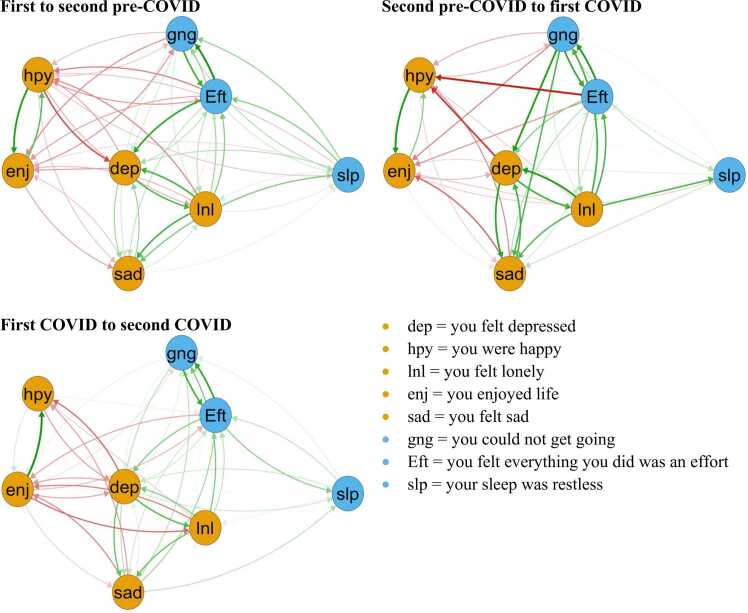
The cross-lagged panel networks for first to second pre-COVID (wave 8: May 2016 – June 2017 → wave 9: June 2018 – July 2019), second pre-COVID to first COVID (wave 9 → COVID-19 wave 1: June – July 2020) and first COVID to second COVID (COVID-19 wave 1 → COVID-19 wave 2: November – December 2020) time-points. The relationship of the CES-D-8 items is indicated by the arrow’s color (green = positive, red = negative) and the strength of the relationship is indicated by the arrow’s thickness (thicker = stronger). The placement of nodes is based on the average layout function in R, only for visualization purposes; the proximity of nodes does not indicate the strength or nature of their relationship. Autoregressive effects are excluded from the graphical representation for a clearer visualization of the cross-lagged effects (yet, autoregressive effects were calculated for each network). All networks with autoregressive paths can be found in Supplemental Material S18 and S19. The items *were happy* and *enjoyed life* were reverse coded. Final N = 7223.

Testing for significant edge weights differences, the edge weights difference tests revealed significantly stronger connections between these edges compared to other edges in the networks (see Figures s7-s9 in the supplemental material) (Fig. 1).

### Symptom centrality

**Fig. 2:**
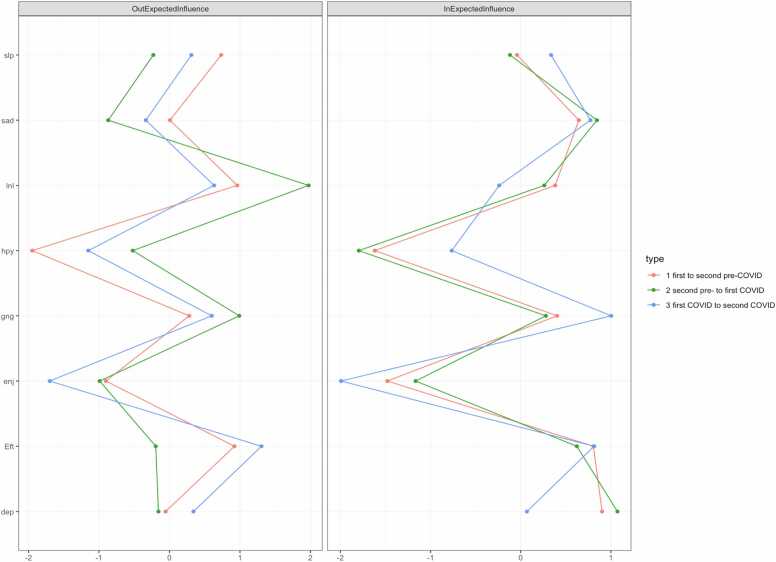
Symptom centrality estimates for the networks using z-values. Greater values indicate greater centrality. Out-expected-influence is the degree to which a symptom predicts other symptoms at the subsequent relevant point. In-expected-influence is the degree to which a symptom is predicted by other symptoms at the subsequent relevant point.

We calculated the out-expected-influence and the in-expected-influence as standardized centrality parameters (see Figures s10-s15 in the supplemental material). These parameters revealed fairly consistent patterns over time, as can be seen in Fig. 2: We found consistently high in-expected-influence for *could not get going* and *everything was an effort*. For the first and the second network, *feeling depressed* had strong incoming connections as well. Overall, also *feeling sad* revealed strong incoming connections over time. Lower in-expected-influence was found for *restless sleep* and *feeling lonely*. Consistently low in-expected-influence was found for the reverse coded symptoms *enjoy life* and *happiness*. For the out-expected-influence, we found high values for *feeling lonely, could not get going* and *everything was an effort* across all networks. *Feeling depressed* and *restless sleep* had weak outgoing connections while *enjoy life* and *happiness* revealed significantly low results.

## Discussion

Using a longitudinal dataset of the UK population aged over 50 years, we aimed to examine the impact of a maximum stressor (i.e., the pandemic) and a repeated stressor (i.e., timepoints during the pandemic) on dynamics of symptoms of depression in older people. Specifically, we used cross-lagged panel network analyses to understand nuances of symptom interrelations among older people. The present findings revealed that, despite the unprecedented stressors of the COVID-19 pandemic, the overall structure of depressive symptom dynamics in older adults remained relatively stable.

While common stressors among older people impose a general burden on this population (e.g., role changes, decreased mobility, or loss; [12]), the pandemic as a unique and prolonged stressor led to additional challenges, particularly during the early phase before widespread availability of vaccines. The population group of older people was confronted with complex stressors such as social isolation, health risks and disruptions in daily routine [10]. During the period of data collection (June–July 2020 and November–December 2020), the UK implemented lockdowns and social distancing measures, which likely amplified these stressors. During the first period, the UK implemented strict lockdown measures including restrictions on non-essential travel, closure of non-essential businesses, and limitations on social gatherings. During the second period, some measures remained in place, but additional restrictions were imposed due to rising COVID-19 cases, such as tighter limits on social contacts, school closures in some regions, and enhanced travel restrictions [52]. This resulted in a higher stringency index during November–December 2020, reflecting greater governmental intervention and stricter limitations compared to the first period (see s20). These phase-specific variations may have contributed to how depressive symptom dynamics in older adults changed across the beginning of the COVID-19 pandemic. Core symptoms such as *could not get going* and *everything was an effort* continued to exhibit strong in- and outgoing connections during the beginning of the COVID-19 pandemic. These findings build on previous findings from Schlechter et al. [9], which identified *everything was an effort, could not get going* and *feeling lonely* as key symptoms in older people, highlighting potential somatic pathways and loneliness in the development of depression networks in older people. The central symptoms identified in our study reflect typical symptoms of depression rather than diagnostic core of major depressive disorder. Our network approach does not assess diagnostic thresholds but instead provides insight on how individual symptoms of depression interrelate and influence each other over time.

We also observed some shifts in symptom interactions, finding *restless sleep* and *feeling lonely* to display lower ingoing but higher outgoing connections during the pandemic, suggesting their increased role in influencing other symptoms of depression under prolonged stress. These changes may reflect the impact of social restrictions and lockdowns in the UK during the early pandemic period. This indicates that these symptoms may act as initiators of depressive symptom cascades under conditions of prolonged stress. From the pre-pandemic to the pandemic period, loneliness emerged as an even stronger predictor of other symptoms of depression under repeated stress, in line with previous research [23], [36], [53]. The observed increase in outgoing connections of loneliness during the pandemic suggests a heightened impact on other symptoms of depression under conditions of prolonged isolation. Consequently, symptoms related to lack of drive, such as *could not get going* and *everything was an effort*, remained activated in the networks during the beginning of the COVID-19 pandemic and played a key role in shaping depressive symptom networks. Additionally, the influence of loneliness underscores the interplay between psychosocial and motivational factors in the development of symptoms of depression in older adults [54]. Theoretical models of loneliness in later life emphasize the role of insufficient social contact and a subjective sense of emotional disconnection, which has been shown to increase affective and motivational symptoms through chronic stress responses [55]. This finding is particularly relevant in the context of government policies that are associated with a reduction in social contact, less activity and a greater need for personal motivation [56]. Over time, these acute challenges evolved into chronic stressors, including prolonged isolation and ongoing health risks, which are known to sustain and exacerbate symptoms of depression [57].

### Clinical implications

Understanding the centrality and stability of symptoms of depression is crucial to inform studies that aim to develop targeted and efficient interventions. Our research showed that the symptoms *everything was an effort* and *could not get going* play a central role in depressive symptom networks of older people. This suggests that therapeutic approaches that target these symptoms could not only alleviate these specific symptoms but could also reduce cascading reactions in the entire symptom network, potentially leading to a general reduction of overall depression symptom severity. In addition, *loneliness* emerged as the symptom with the highest out-expected influence in the pre-pandemic network, predicting depressive symptom changes under acute stress. This finding underscores the importance of addressing psychosocial factors, particularly loneliness, in therapeutic approaches. Interventions to increase social contact and reduce isolation can not only reduce loneliness itself but also mitigate its impact on other symptoms of depression deterioration, especially under acute or chronic stress conditions.

Cognitive-behavioral strategies aiming at behavioral activation could be of particular interest [58]. By increasing the activity level of patients, an improvement in their general mental well-being may be achieved. As current research shows, behavioral activation has diverse positive effects on symptoms of depression leading to a reduction of feeling depressed, changes in weight and appetite, concentration, and psychomotor agitation or retardation [59]. Thus behavioral activation can lead to a reduction in general network connectivity, resulting in an upward positive spiral [60]. This finding is especially relevant as older people often suffer from physical inactivity and social withdrawal, representing risk factors in the development and maintenance of depression [16]. In addition, previous studies have shown that behavioral activation leads generally to a significant reduction in symptoms of depression [61] as well as a decrease in emotional loneliness during the COVID-19 pandemic [62].

Our findings suggest that somatic symptoms like *could not get going* and *everything was an effort* are central in depression networks of older adults, while the DSM-V hallmark symptom *feeling depressed* and *feeling sad* [35] rather represent an end product of a symptomatic process. Additionally, loneliness emerged as a symptom with high out-expected influence, predicting further symptom cascades and playing a key role in the progression of depressive networks. This indicates that early symptoms of depression in older adults may differ from the typical DSM-5 diagnostic criteria for major depressive disorder [35], which focus primarily on affective and cognitive symptoms such as persistent depressed mood or anhedonia, emphasizing the need to consider somatic symptoms and loneliness in the diagnostic process. Given the underdiagnosis and undertreatment of depression in this age group [63], it is crucial to recognize these symptoms as early as possible. For clinicians, it is important to distinguish somatic symptoms within the context of age-related changes [64]. These findings suggest that older adults reporting a persistent lack of energy, difficulties in getting it going, or increased loneliness may be at heightened risk for developing or worsening symptoms of depression. During the early stages of the COVID-19 pandemic, these symptoms were particularly influential in driving other symptoms of depression, highlighting potential targets for early screening and preventive interventions. Addressing these symptoms proactively could support mental health and well-being in older adults, especially under conditions of prolonged stress.

### Strengths and limitations

Our study addresses a relevant gap in understanding how depressive symptom networks in older adults respond to acute and prolonged stressors as the COVID-19 pandemic. One key strength of our study is the use of a longitudinal design, which allows us to observe temporal trends in symptoms of depression across multiple timepoints. In addition, the network analytical approach allows us to gain nuanced insights into the interrelation and centrality of specific symptoms.

However, there are limitations to our study. The generalizability of our findings may be limited due to the specific demographic and geographic characteristics of our sample. Our sample might not adequately represent the diversity of older people within the UK as well as outside the UK. For instance, ELSA participants are disproportionately white, individuals from lower socio-economic groups and those experiencing severe health issues were more likely to drop out of the study [9].

In addition, the fact that we relied on self-report measures could lead to a bias as respondents may underreport their symptoms or misinterpret them. Older people may underreport their symptoms, either due to trivialization of their distress or misinterpreting psychological distress as somatic symptoms. Employing a combination of self-report data and external assessments, e.g., by structured clinical interviews, could provide a more comprehensive understanding of symptoms of depression in older populations.

In addition, the dichotomous format of the CES-D-8 items may limit the overall sensitivity of responses as it does not capture different levels of symptom severity. This limits the ability to distinguish between mild, moderate, and severe symptoms of depression. Future research could potentially benefit from using scales with more nuanced response options to better reflect the complexity of depressive experiences in this population. Moreover, the CES-D-8 assesses only eight symptoms over a one-week period, which may not fully capture the breadth or duration of depressive experience. Future research could benefit from using more comprehensive symptom scales covering a wider range of symptoms of depression and longer time frames to better reflect the complexity of depressive experience in this population.

Concerning the CLPN analysis, a bias could be its inability to disentangle between-person from within-person effects [43]. Furthermore, the present data were collected for COVID-19 wave 1 from June to July 2020 and for COVID-19 wave 2 from November to December 2020. During these periods, the UK experienced national lockdowns, social distancing, and other public health measures aimed at controlling virus spread. Regarding the COVID-19 new cases in the UK, incidence peaks were found to take part in April 2020, in November 2020, in January 2021, and in July 2021. Therefore, a more fine-grained analysis as a function of COVID-19 incidence and public health emergency measures was not possible due to the lack of available data during these times. In addition, the first COVID-19 wave in the ELSA dataset does not match the first COVID-19 incidence peak, potentially preventing us from detecting effects during the start of the pandemic.

## Conclusion

Our study contributes to the understanding of the dynamics of symptoms of depression in older people during the COVID-19 pandemic. Our findings underline the stability of depressive symptom networks over time, with *could not get going*, *everything was an effort* and *loneliness* as core symptoms over time. We show that symptoms of depression network during the COVID-19 pandemic were similar to pre-pandemic networks. Our findings have the potential to inform studies that develop targeted mental health interventions and support public health strategies for mental health and wellbeing of older people during a pandemic.

## Declaration of Competing Interest

The authors declare that they have no known competing financial interests or personal relationships that could have appeared to influence the work reported in this paper.
