# Supplementary material for: Temporal depressive symptom networks in older adults during the COVID-19 pandemic
Source: J Mood Anxiety Disord. 2025 Nov 7;12:100156. doi: 10.1016/j.xjmad.2025.100156 (PMC12657744; doi:10.1016/j.xjmad.2025.100156)

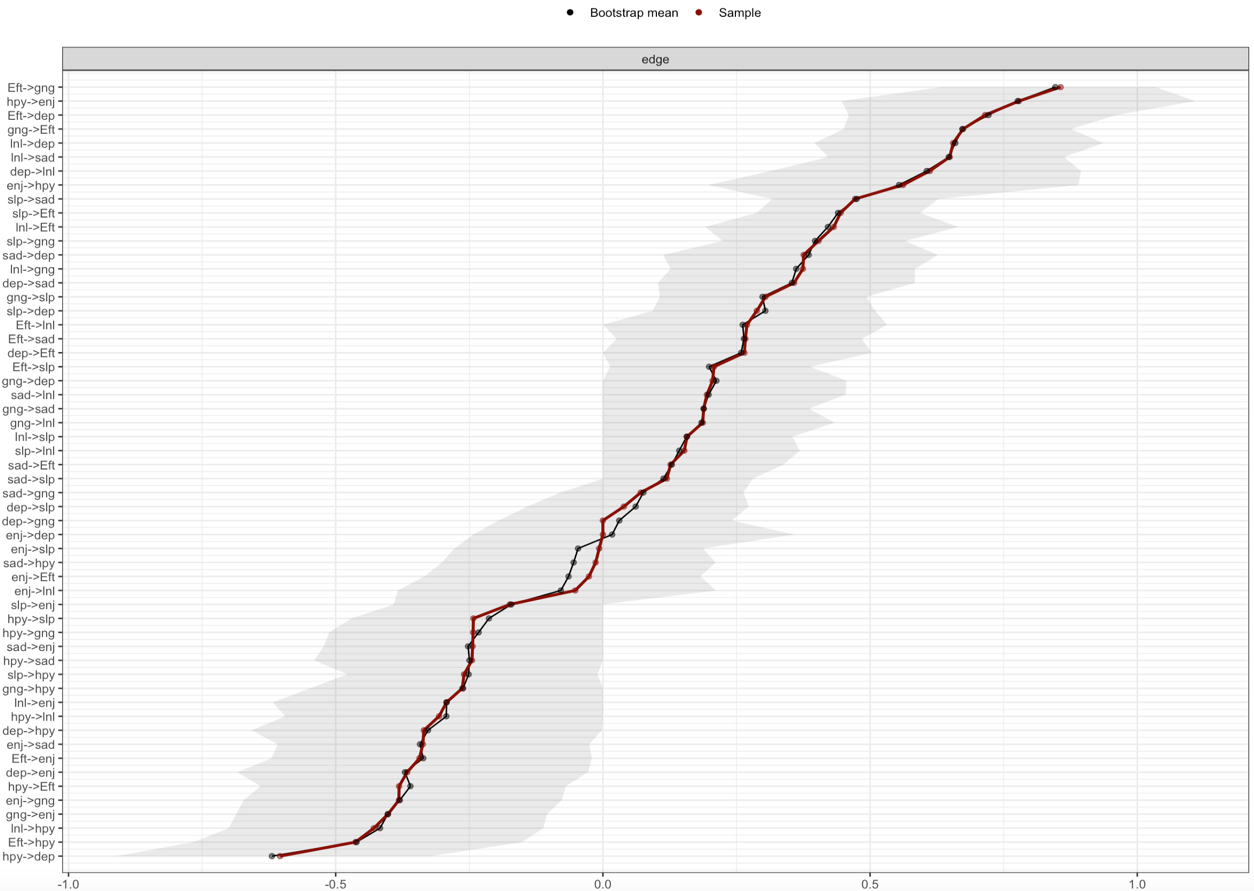


**Figure s1.** CES-D-8 item interrelation accuracy plots for the first Pre-COVID (T1) → second Pre-COVID (T2) network with 1000 bootstrap iterations. The plot shows the sample interrelations (i.e., edge weights; red dots), the means of the bootstrapped interrelations (i.e., edge weights; black dots), and the bootstrap confidence intervals. Reverse coding was applied to the CES-D-8 items *you were happy* and *enjoying life*.

dep = you felt depressed

hpy = you were happy

lnl = you felt lonely

enj = you enjoyed life

sad = you felt sad

gng = you could not get going

Eft = you felt everything you did was an effort

slp = your sleep was restless

**
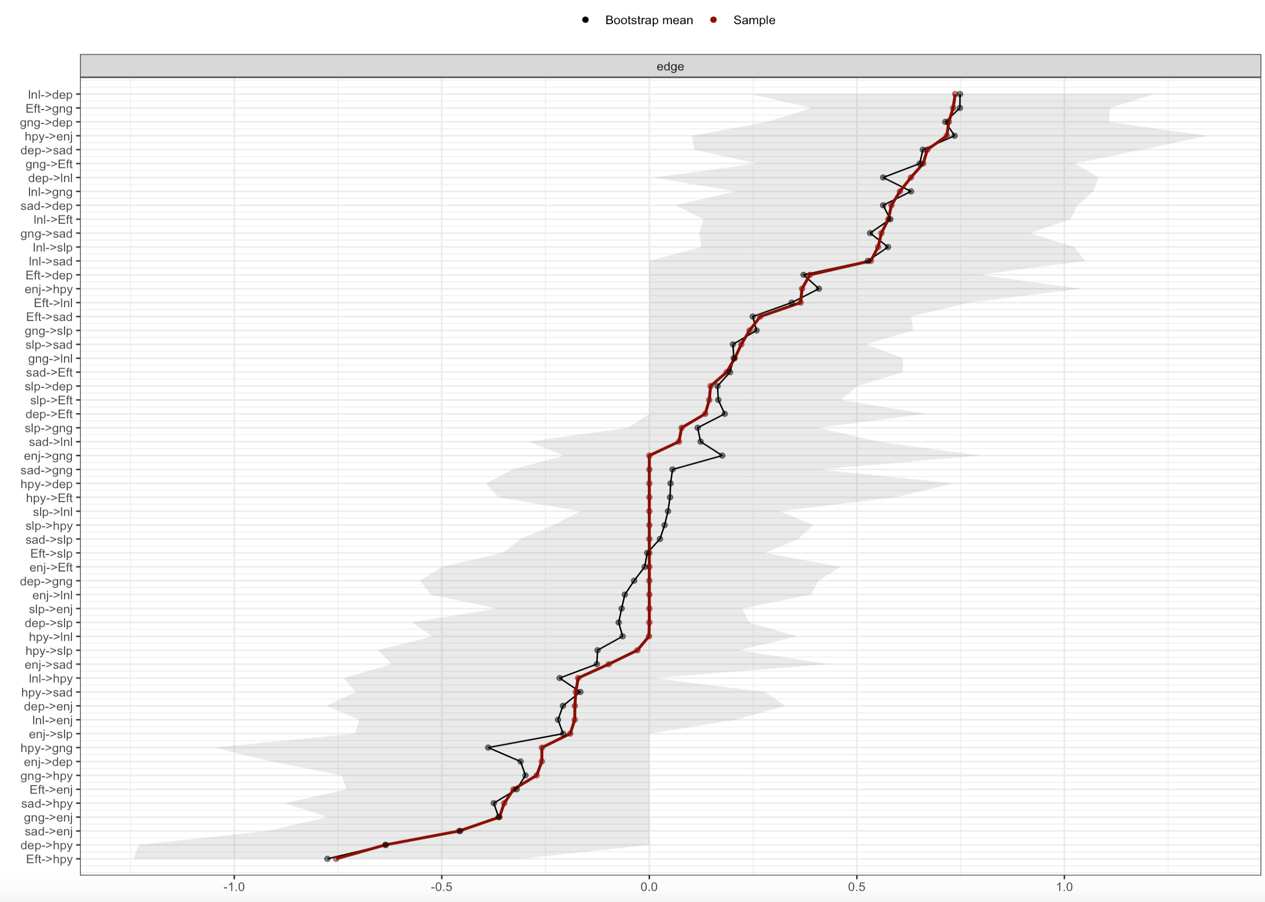
**

**Figure s2.** CES-D-8 item interrelation accuracy plots for the t2 → first COVID-19 (T3) network with 1000 bootstrap iterations. The plot shows the sample interrelations (i.e., edge weights; red dots), the means of the bootstrapped interrelations (i.e., edge weights; black dots), and the bootstrap confidence intervals. Reverse coding was applied to the CES-D-8 items *you were happy* and *enjoying life*.

dep = you felt depressed

hpy = you were happy

lnl = you felt lonely

enj = you enjoyed life

sad = you felt sad

gng = you could not get going

Eft = you felt everything you did was an effort

slp = your sleep was restless


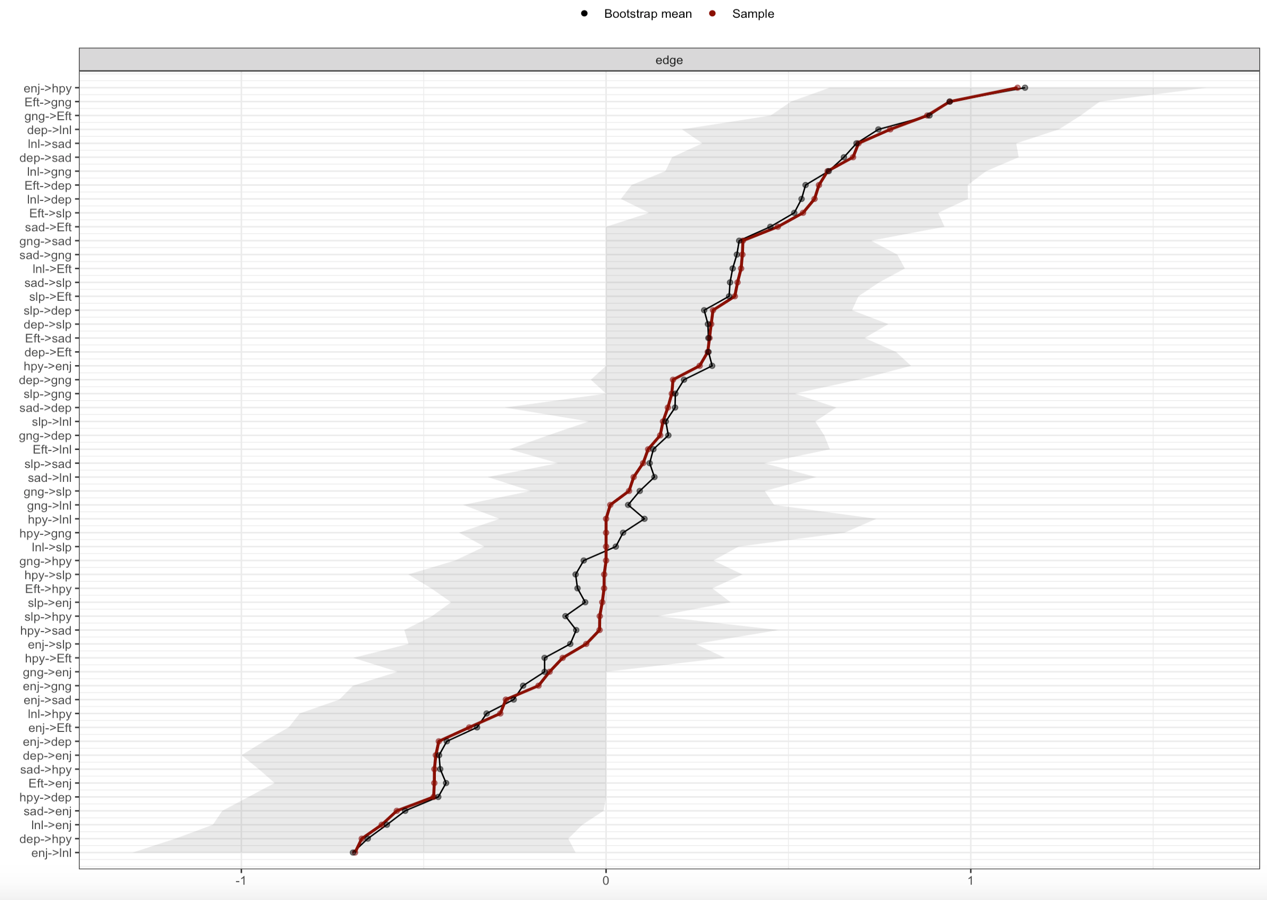


**Figure s3.** CES-D-8 item interrelation accuracy plots for the T3 → second COVID-19 (T4) network with 1000 bootstrap iterations. The plot shows the sample interrelations (i.e., edge weights; red dots), the means of the bootstrapped interrelations (i.e., edge weights; black dots), and the bootstrap confidence intervals. Reverse coding was applied to the CES-D-8 items *you were happy* and *enjoying life*.

dep = you felt depressed

hpy = you were happy

lnl = you felt lonely

enj = you enjoyed life

sad = you felt sad

gng = you could not get going

Eft = you felt everything you did was an effort

slp = your sleep was restless

**
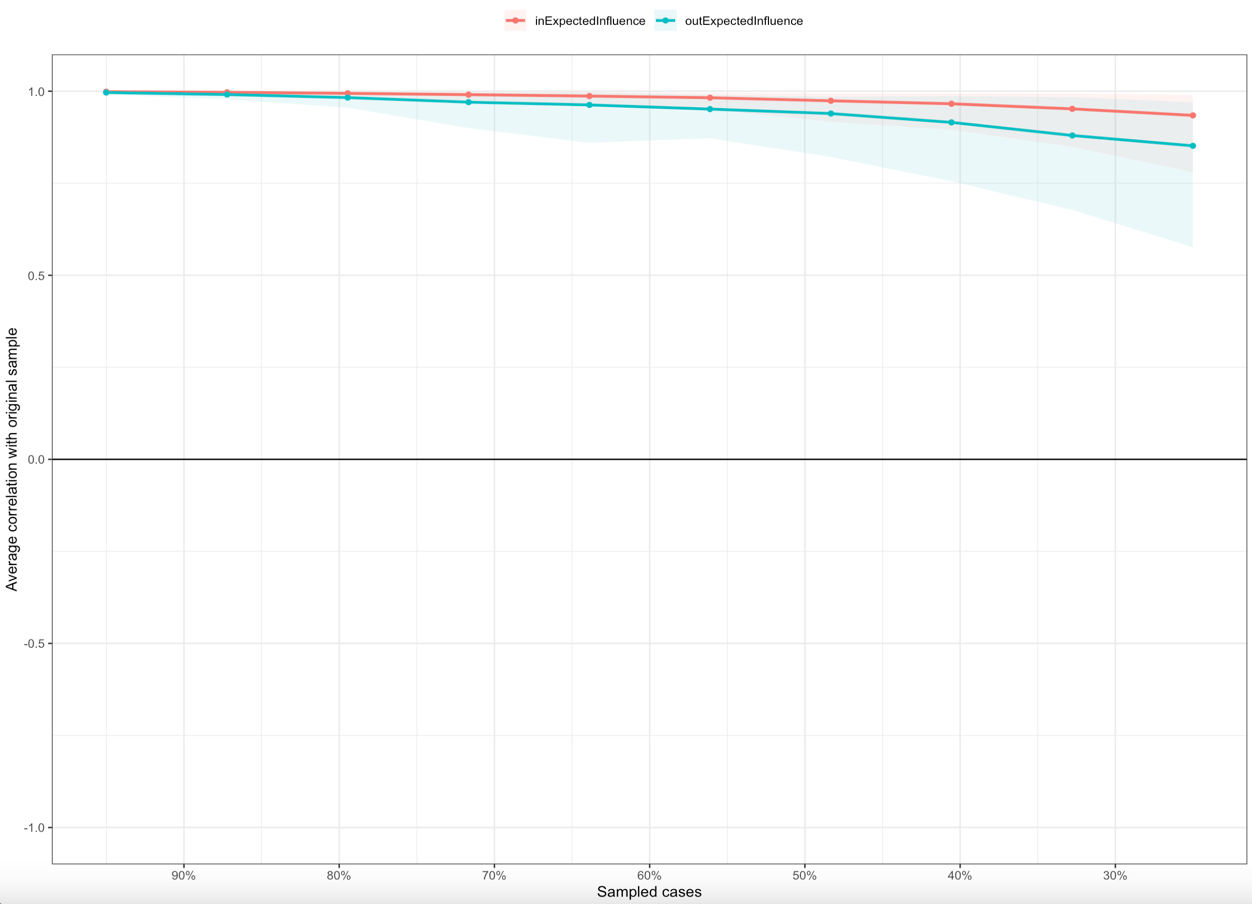
**

**Figure s4.** Stability of centrality measures for the T1 → T2 network


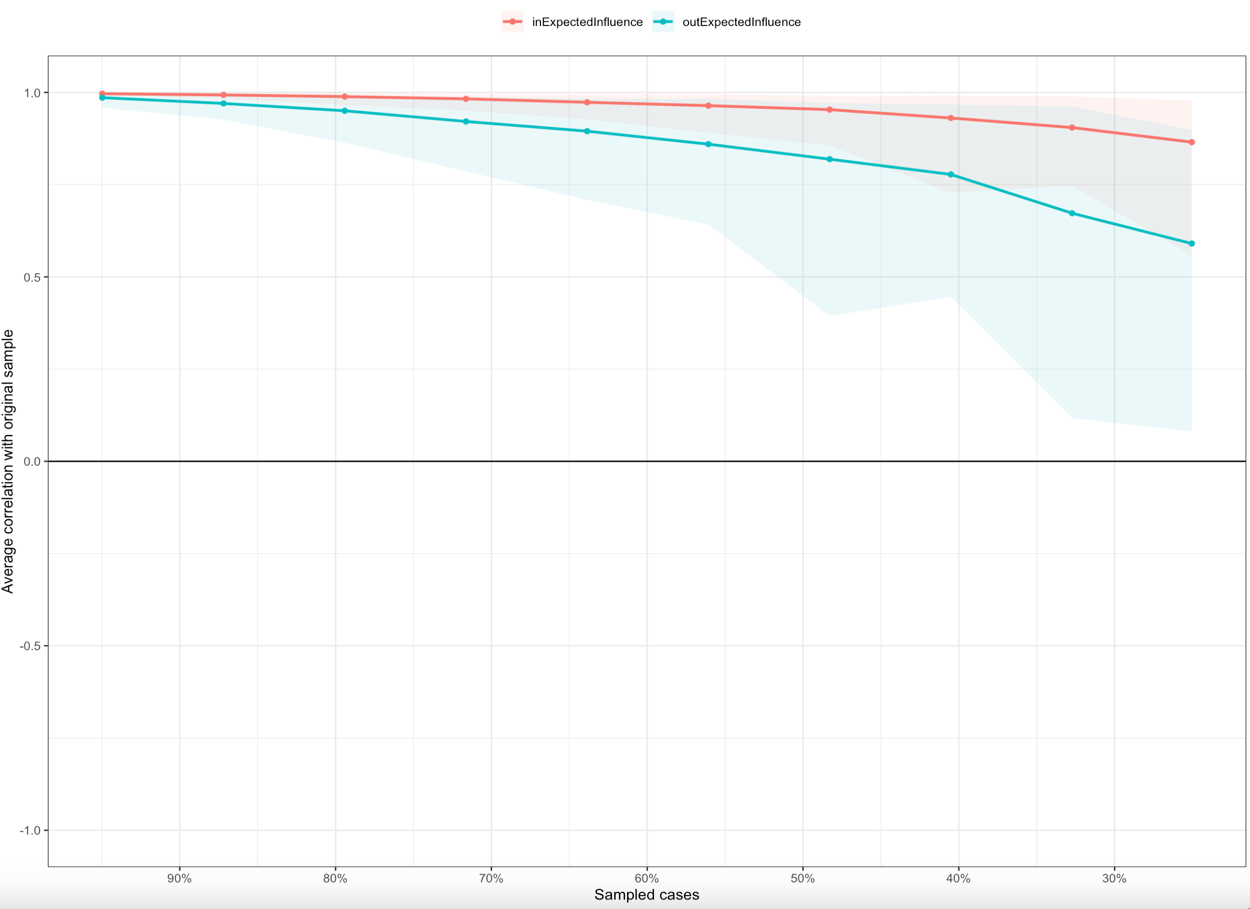


**Figure s5.** Stability of centrality measures for the T2 → T3 network


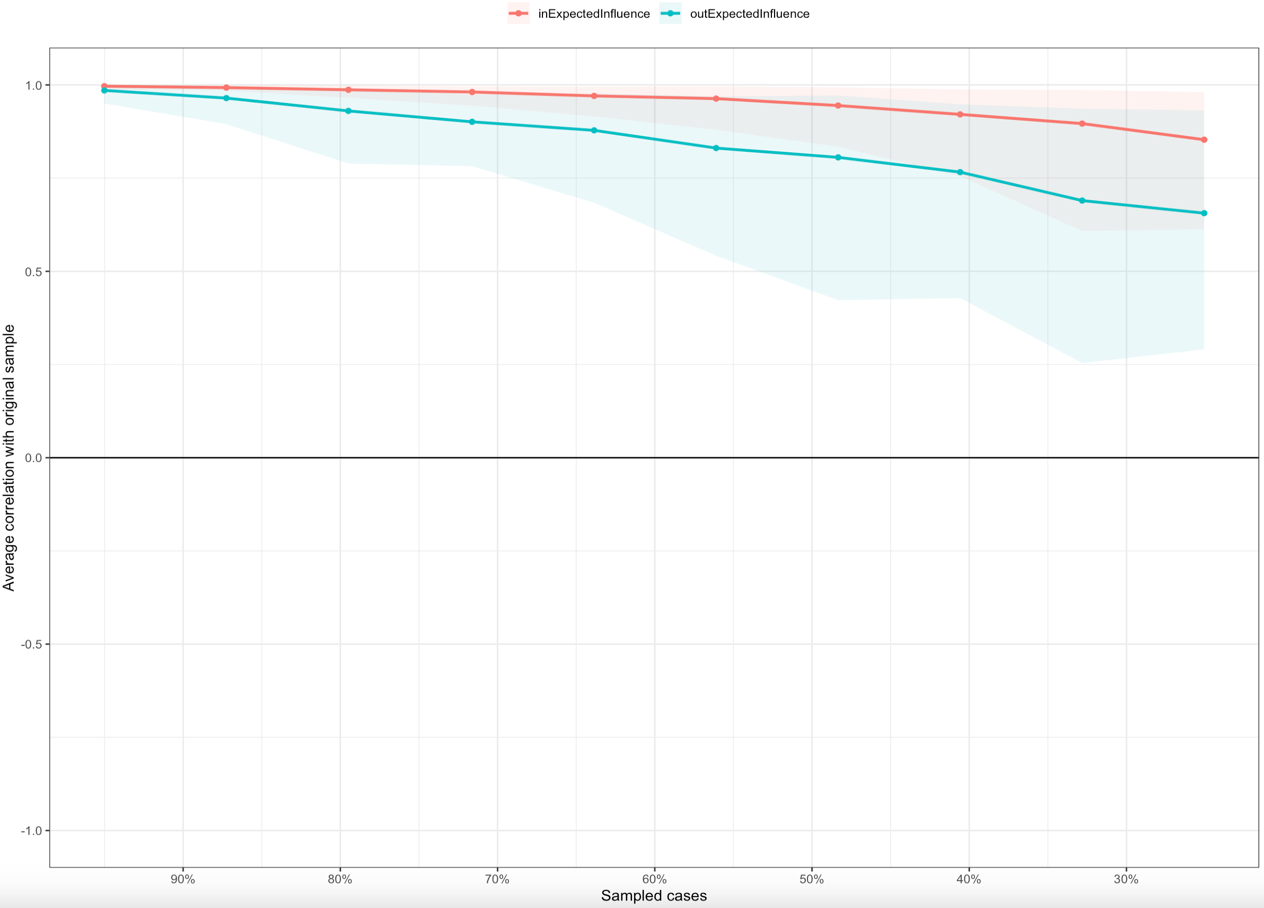


**Figure s6.** Stability of centrality measures for the T3 → T4 network

**
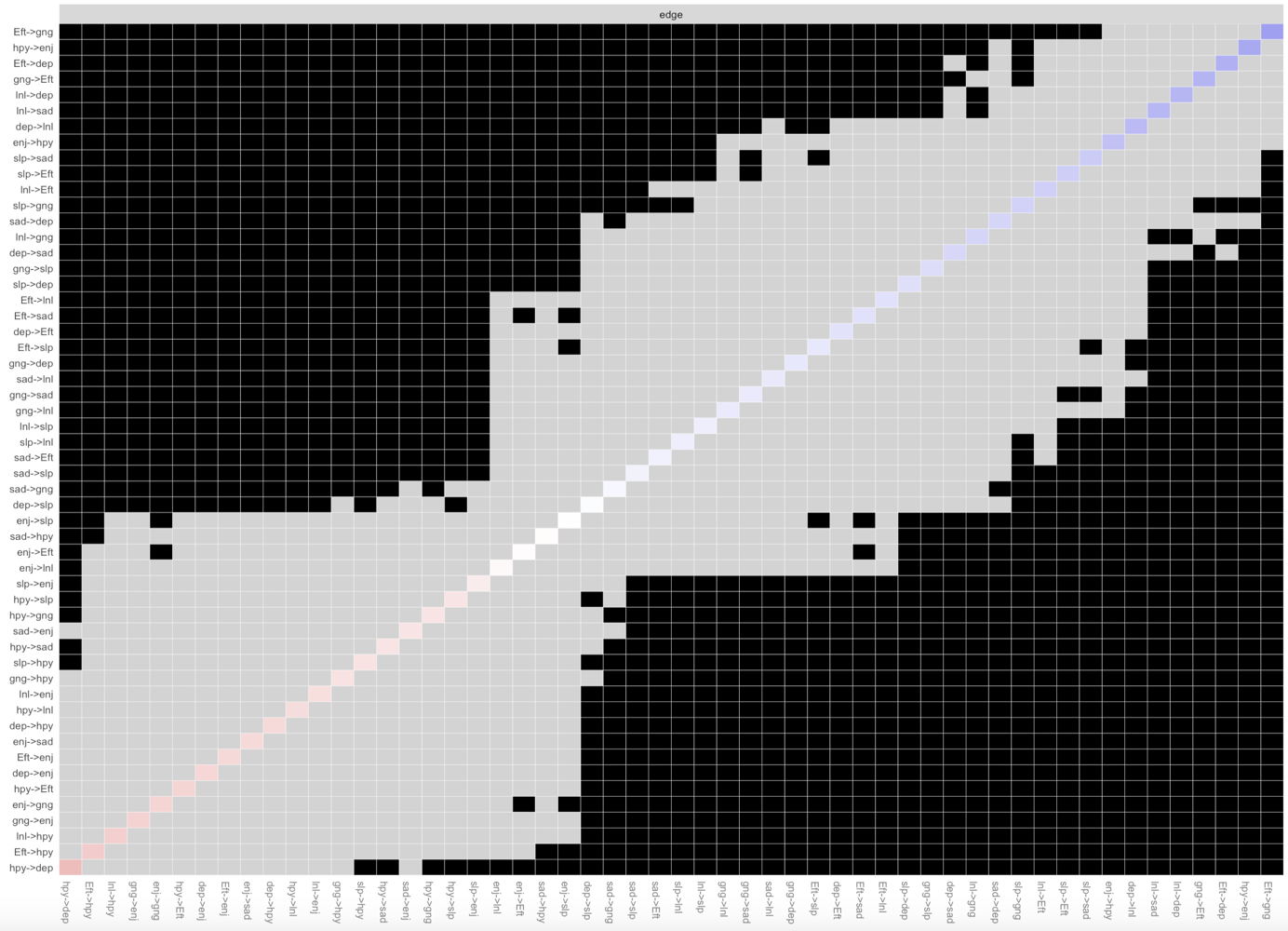
**

**Figure s7.** Edge weight difference tests for the T1 → T2 network with black boxes indicating edges that significantly differ from each other (*p* < .05) and gray boxes indicating no differences. Reverse coding was applied to the CES-D-8 items *you were happy* and *enjoying life*.

dep = you felt depressed

hpy = you were happy

lnl = you felt lonely

enj = you enjoyed life

sad = you felt sad

gng = you could not get going

Eft = you felt everything you did was an effort

slp = your sleep was restless

**
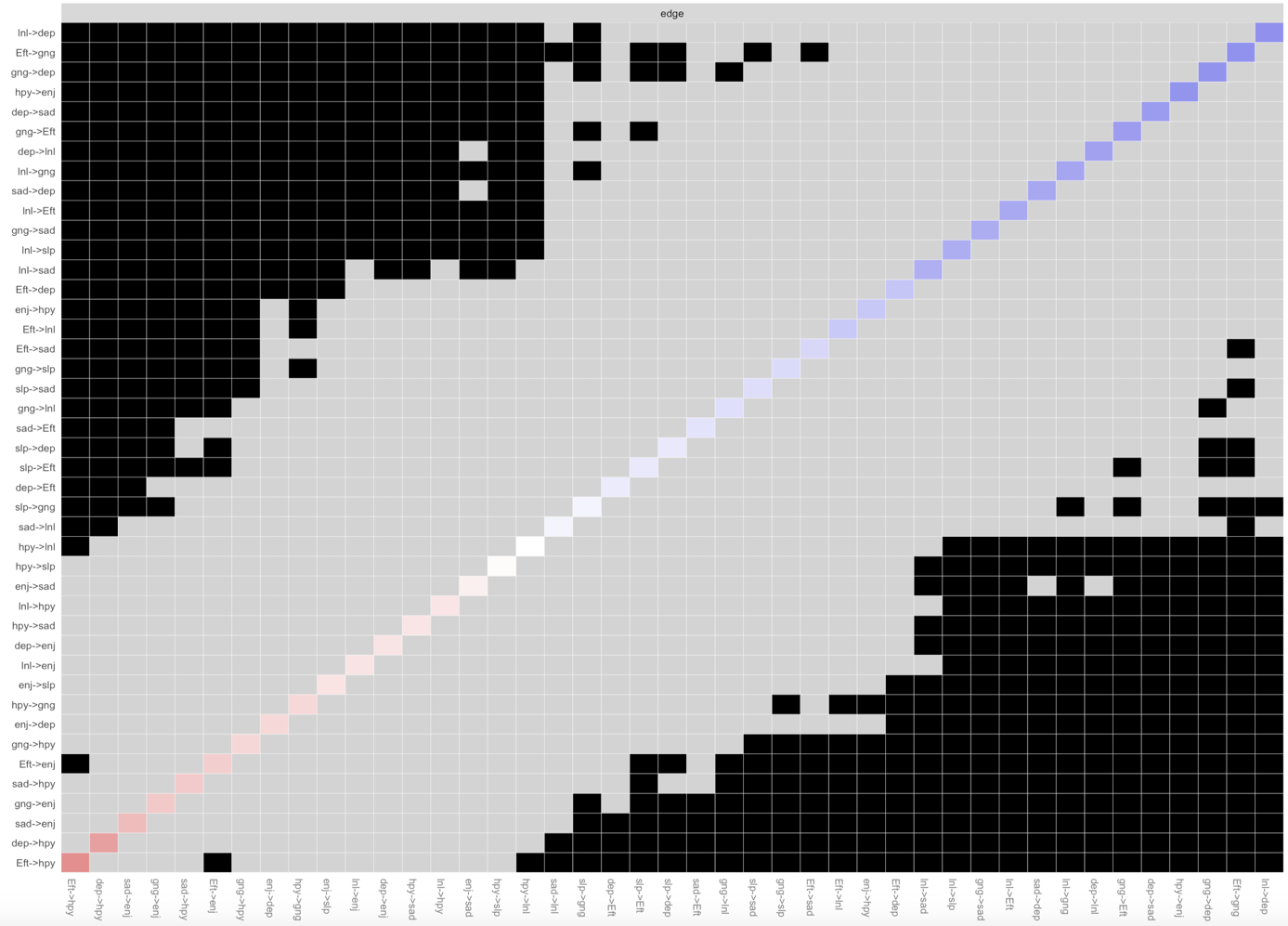
**

**Figure s8.** Edge weight difference tests for the T2 → T3 network with black boxes indicating edges that significantly differ from each other (*p* < .05) and gray boxes indicating no differences. Reverse coding was applied to the CES-D-8 items *you were happy* and *enjoying life*.

dep = you felt depressed

hpy = you were happy

lnl = you felt lonely

enj = you enjoyed life

sad = you felt sad

gng = you could not get going

Eft = you felt everything you did was an effort

slp = your sleep was restless

**
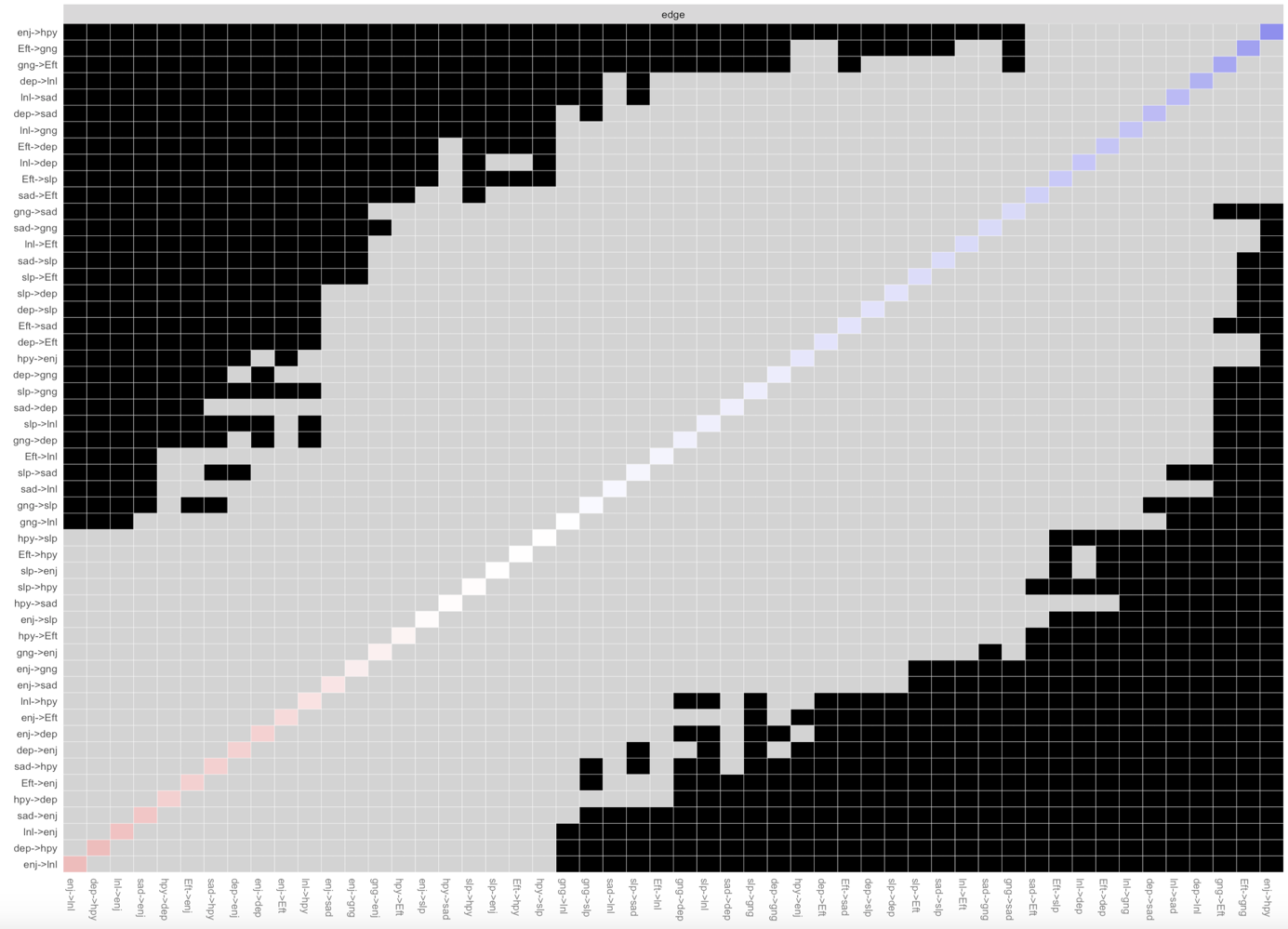
**

**Figure s9.** Edge weight difference tests for the T3 → T4 network with black boxes indicating edges that significantly differ from each other (*p* < .05) and gray boxes indicating no differences. Reverse coding was applied to the CES-D-8 items *you were happy* and *enjoying life*.

dep = you felt depressed

hpy = you were happy

lnl = you felt lonely

enj = you enjoyed life

sad = you felt sad

gng = you could not get going

Eft = you felt everything you did was an effort

slp = your sleep was restless


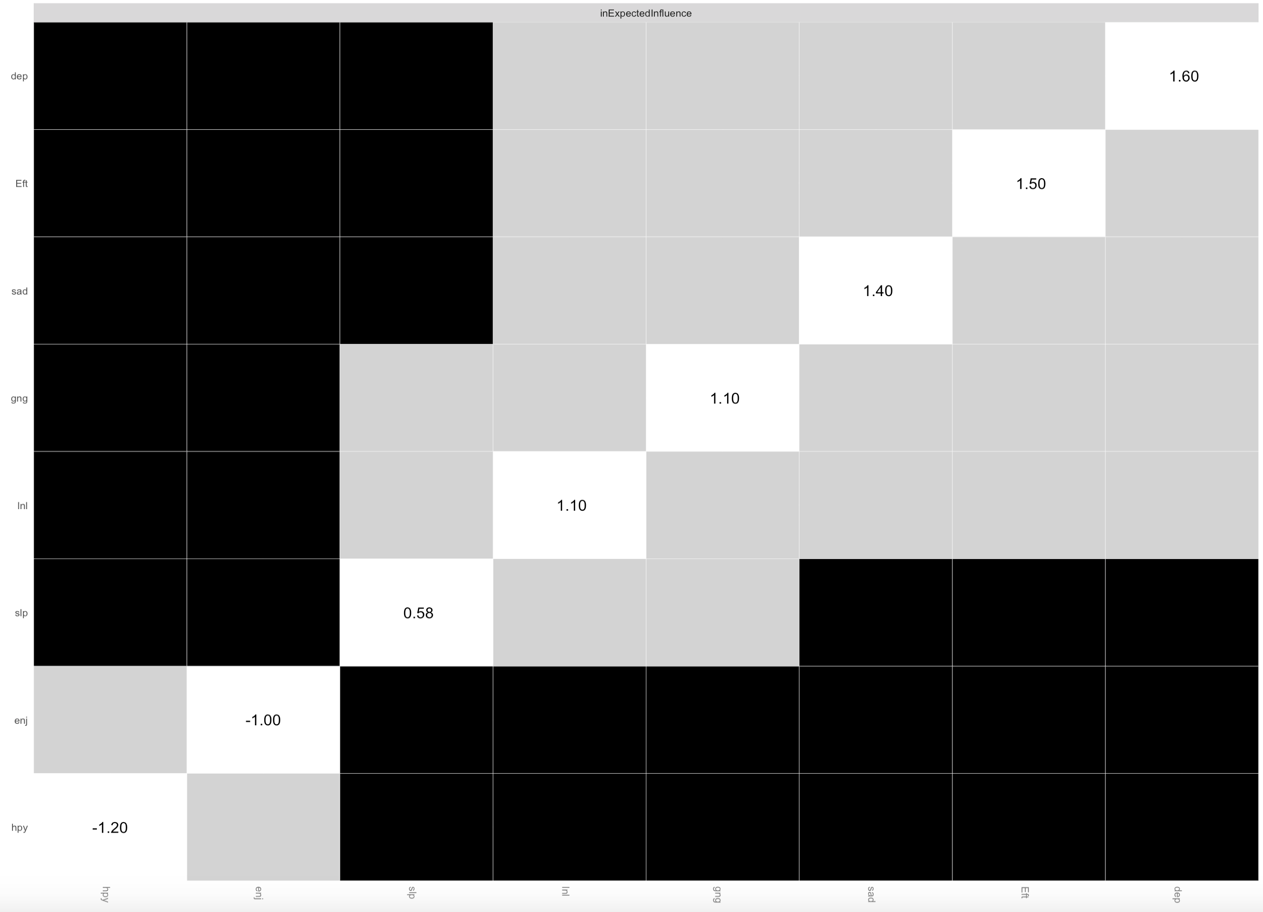


**Figure s10.** In-expected-influence difference tests for the T1 → T2 network with black boxes indicating edges that significantly differ from each other (*p* < .05) and gray boxes indicating no differences. Reverse coding was applied to the CES-D-8 items *you were happy* and *enjoying life*.

dep = you felt depressed

hpy = you were happy

lnl = you felt lonely

enj = you enjoyed life

sad = you felt sad

gng = you could not get going

Eft = you felt everything you did was an effort

slp = your sleep was restless


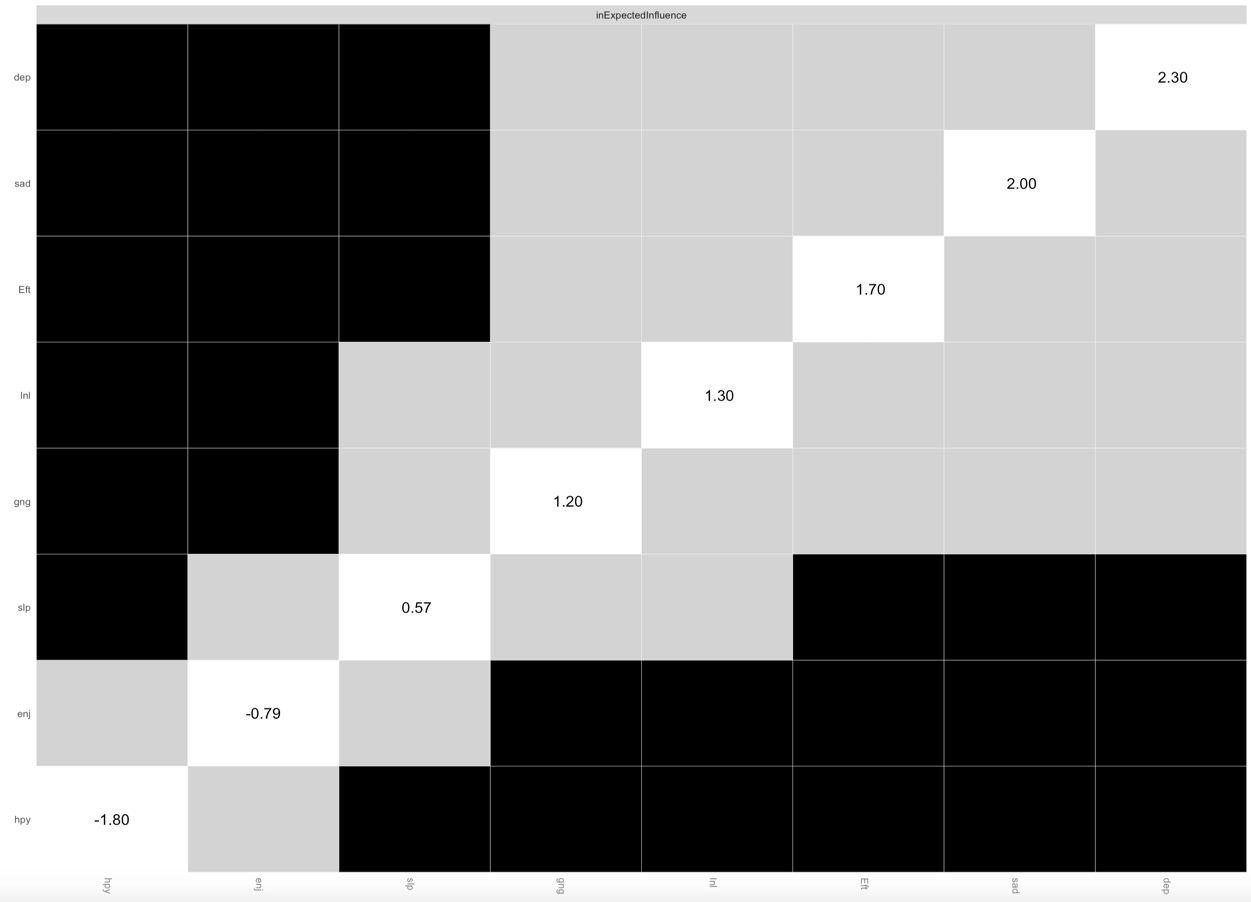


**Figure s11.** In-expected-influence difference tests for the T2 → T3 network with black boxes indicating edges that significantly differ from each other (*p* < .05) and gray boxes indicating no differences. Reverse coding was applied to the CES-D-8 items *you were happy* and *enjoying life*.

dep = you felt depressed

hpy = you were happy

lnl = you felt lonely

enj = you enjoyed life

sad = you felt sad

gng = you could not get going

Eft = you felt everything you did was an effort

slp = your sleep was restless


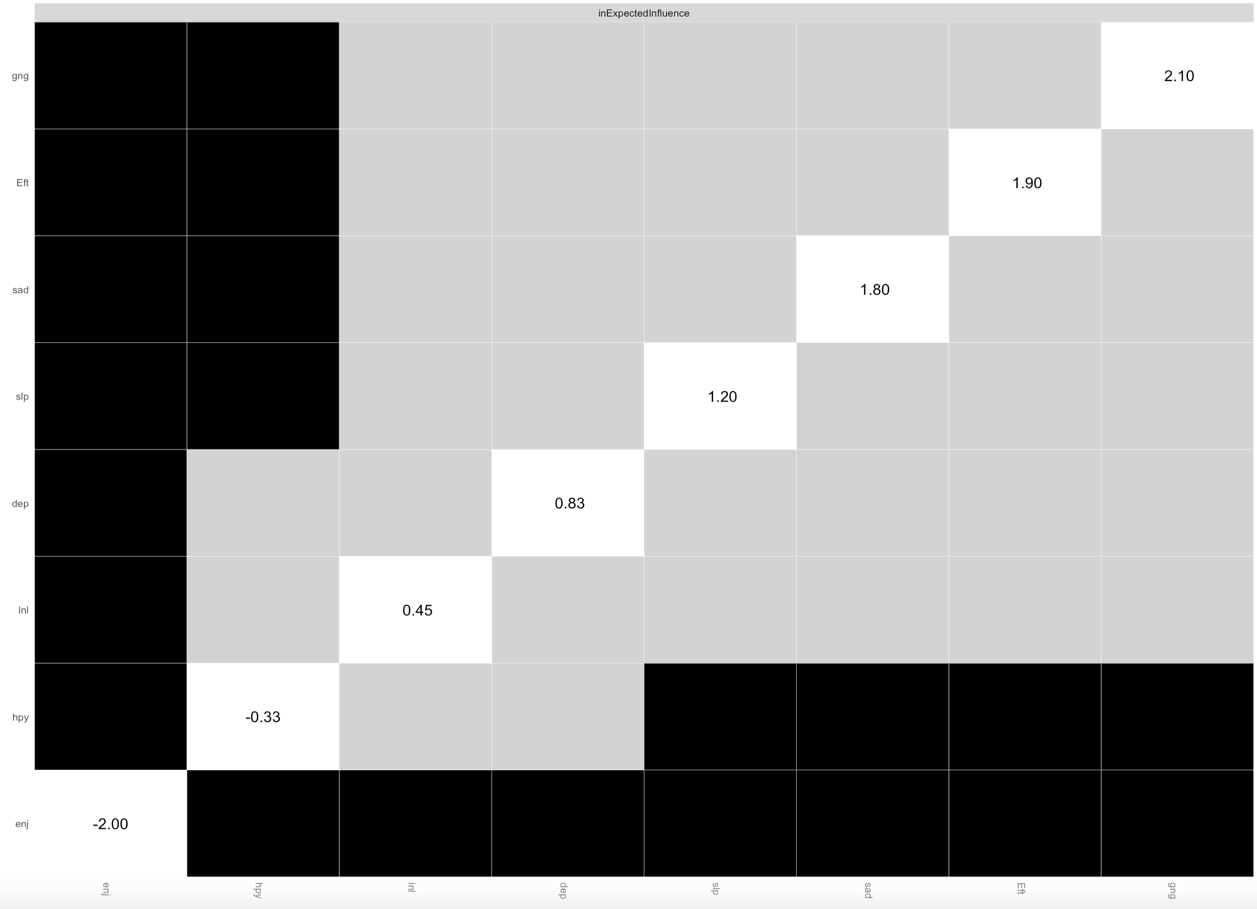


**Figure s12.** In-expected-influence difference tests for the T3 → T4 network with black boxes indicating edges that significantly differ from each other (*p* < .05) and gray boxes indicating no differences. Reverse coding was applied to the CES-D-8 items *you were happy* and *enjoying life*.

dep = you felt depressed

hpy = you were happy

lnl = you felt lonely

enj = you enjoyed life

sad = you felt sad

gng = you could not get going

Eft = you felt everything you did was an effort

slp = your sleep was restless


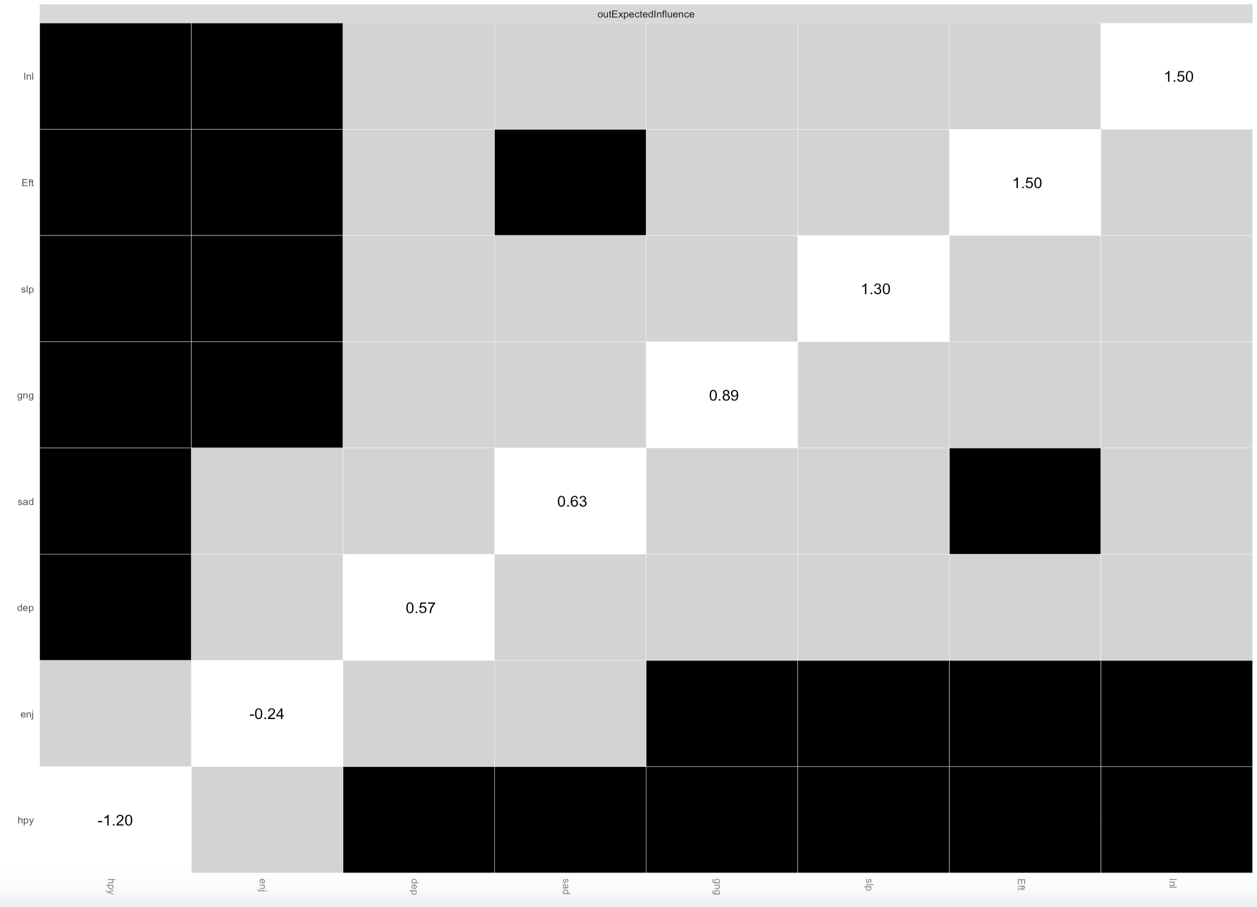


**Figure s13.** Out-expected-influence difference tests for the T1 → T2 network with black boxes indicating edges that significantly differ from each other (*p* < .05) and gray boxes indicating no differences. Reverse coding was applied to the CES-D-8 items *you were happy* and *enjoying life*.

dep = you felt depressed

hpy = you were happy

lnl = you felt lonely

enj = you enjoyed life

sad = you felt sad

gng = you could not get going

Eft = you felt everything you did was an effort

slp = your sleep was restless


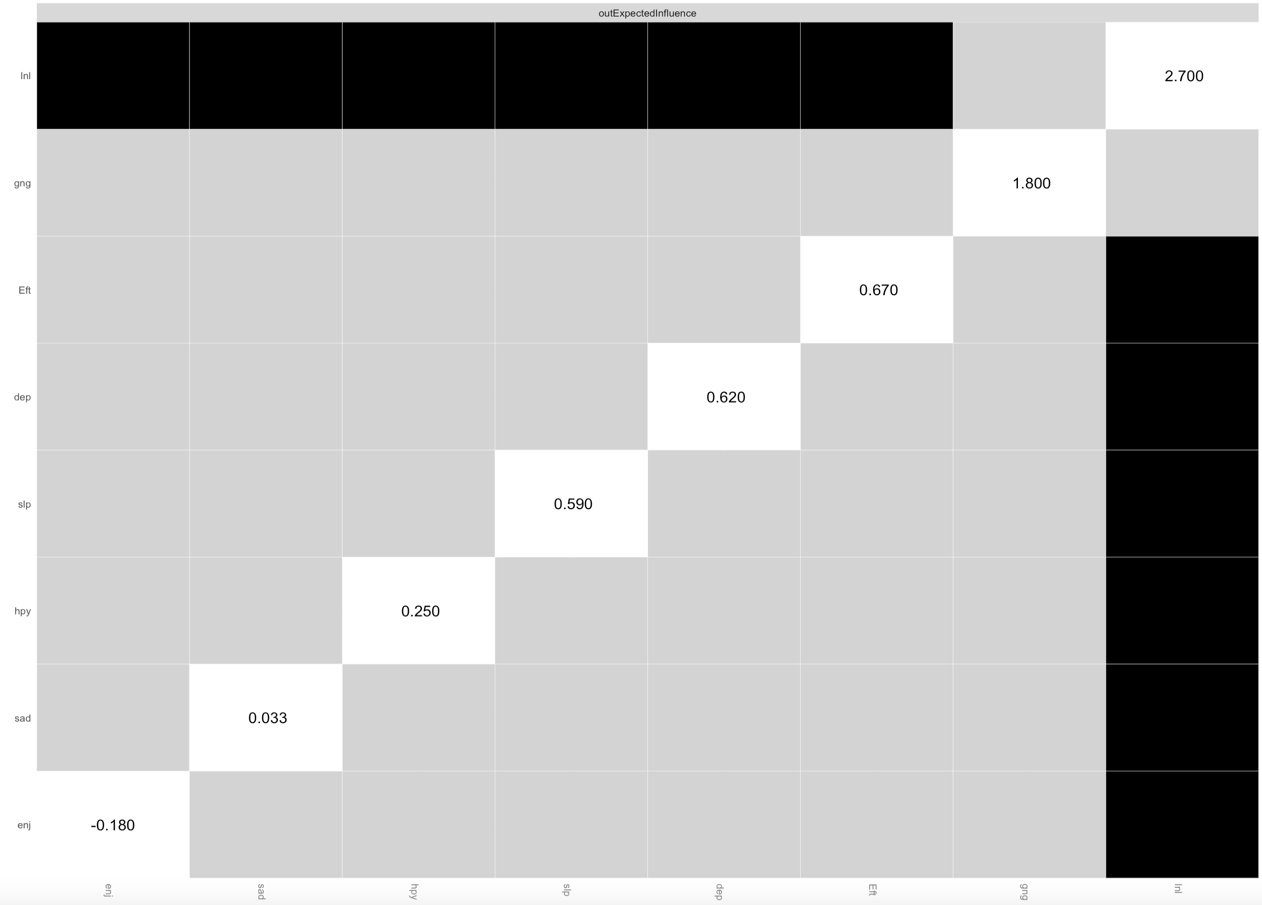


**Figure s14.** Out-expected-influence difference tests for the T2 → T3 network with black boxes indicating edges that significantly differ from each other (*p* < .05) and gray boxes indicating no differences. Reverse coding was applied to the CES-D-8 items *you were happy* and *enjoying life*.

dep = you felt depressed

hpy = you were happy

lnl = you felt lonely

enj = you enjoyed life

sad = you felt sad

gng = you could not get going

Eft = you felt everything you did was an effort

slp = your sleep was restless


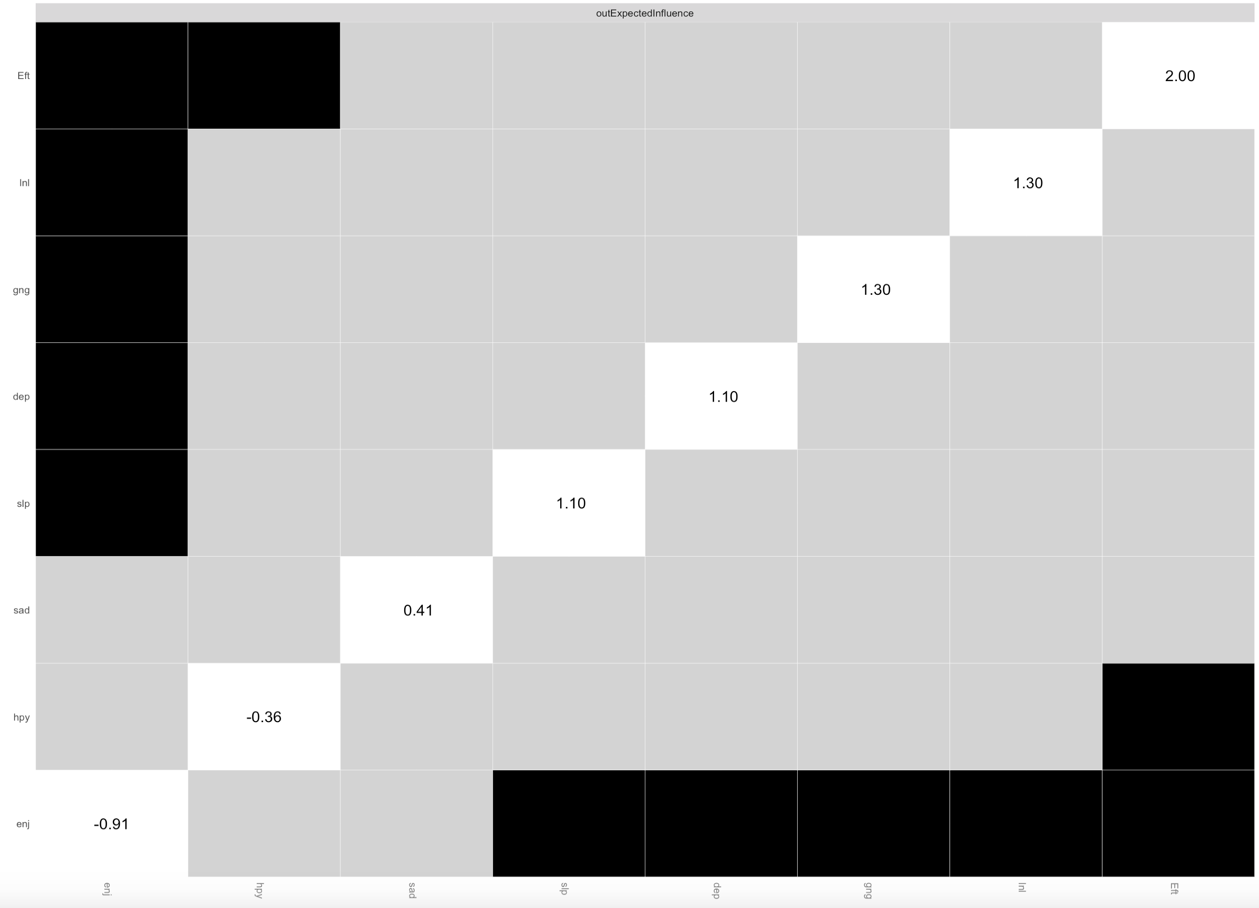


**Figure s15.** Out-expected-influence difference tests for the T3 → T4 network with black boxes indicating edges that significantly differ from each other (*p* < .05) and gray boxes indicating no differences. Reverse coding was applied to the CES-D-8 items *you were happy* and *enjoying life*.

dep = you felt depressed

hpy = you were happy

lnl = you felt lonely

enj = you enjoyed life

sad = you felt sad

gng = you could not get going

Eft = you felt everything you did was an effort

slp = your sleep was restless

| **First to second pre-COVID**  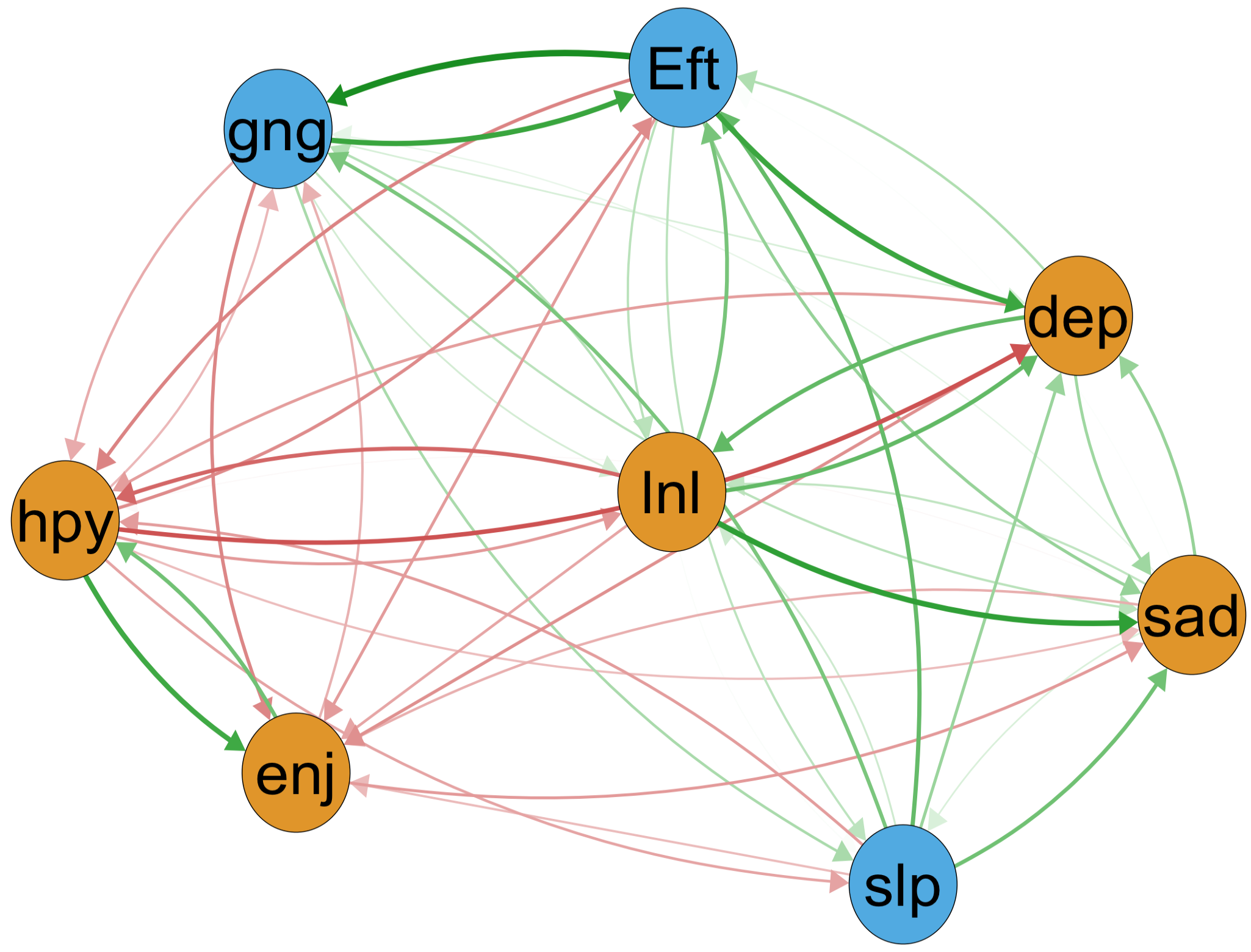 | **Second pre-COVID to first COVID**  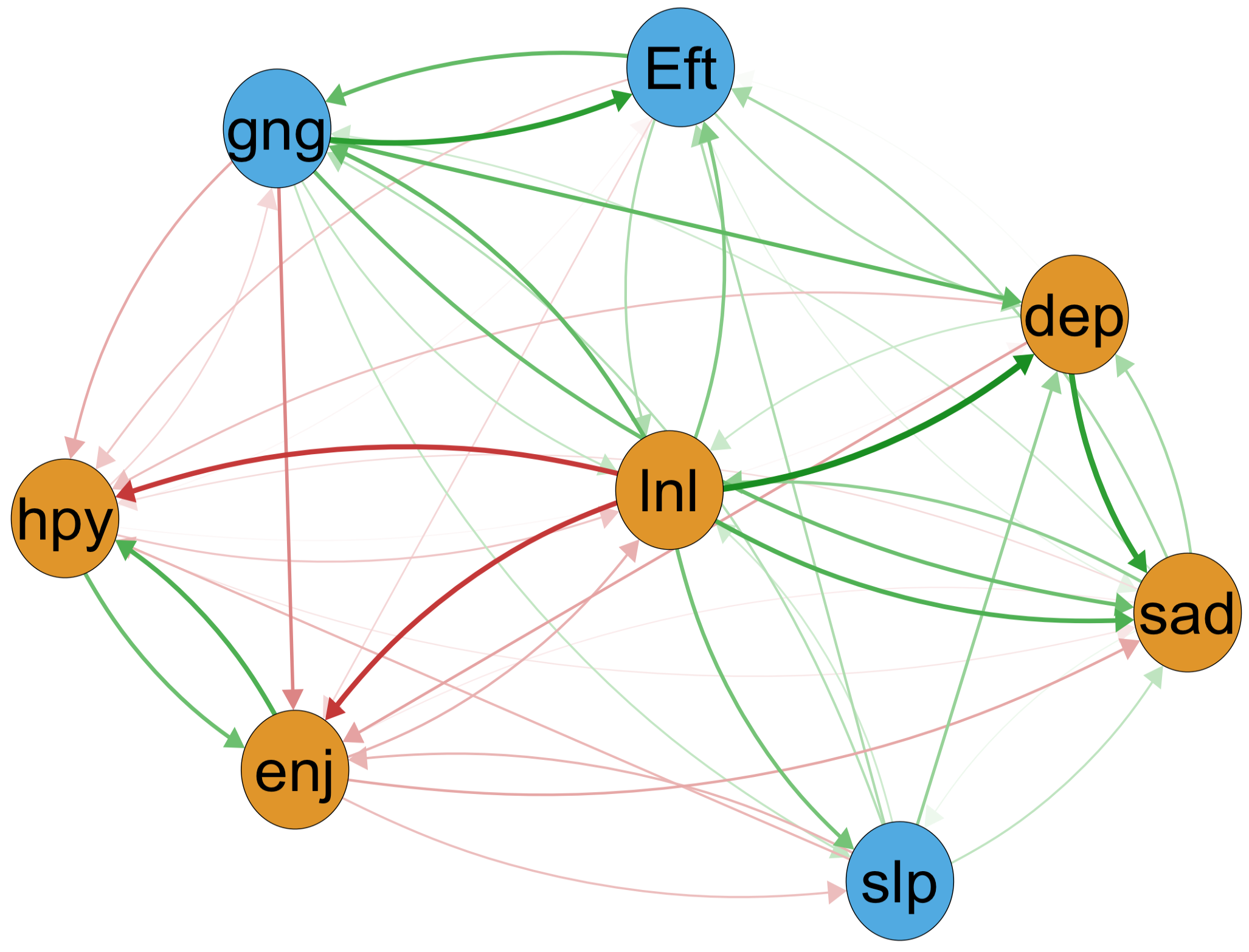 |
| --- | --- |
| **First COVID to second COVID**  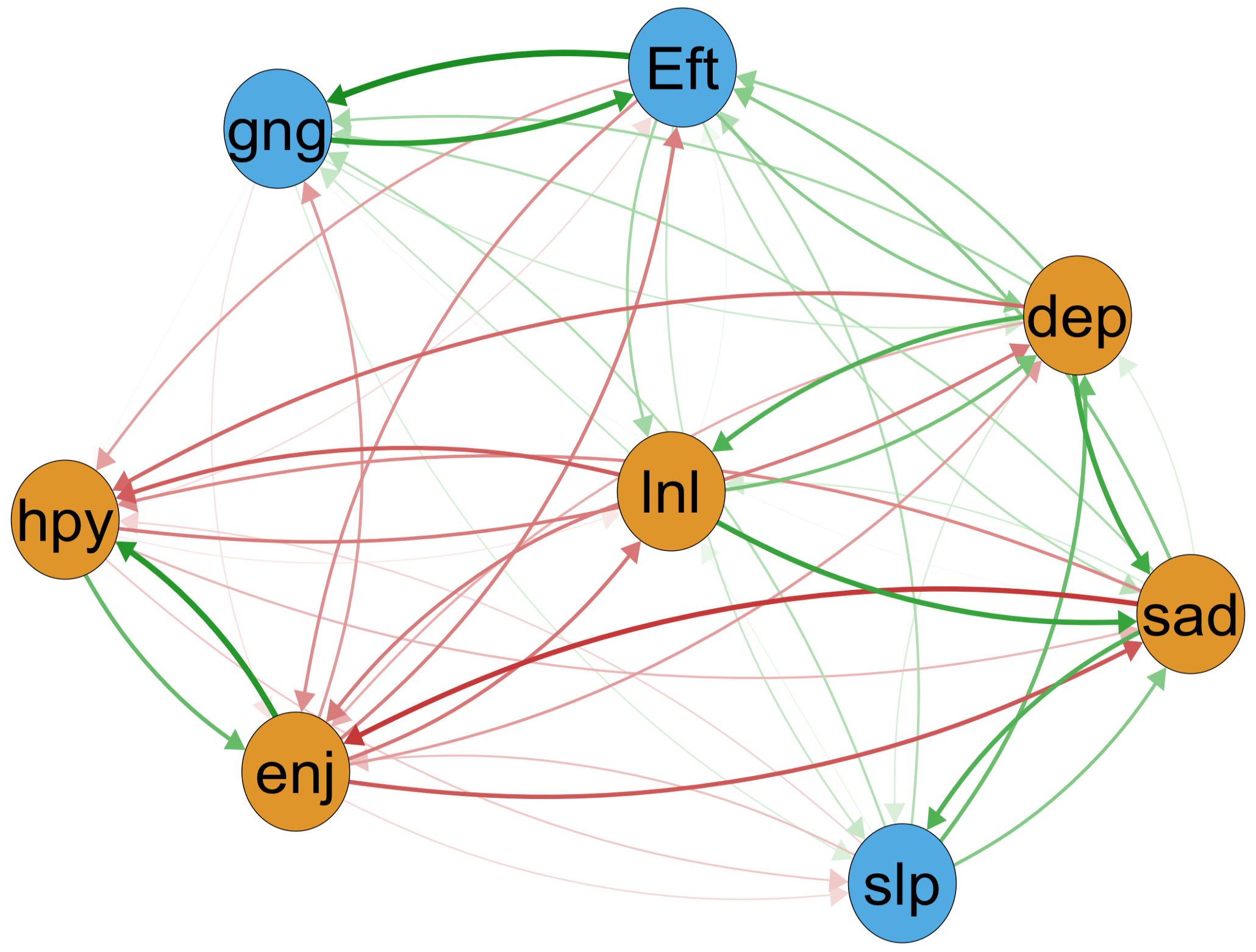 | - dep = you felt depressed - hpy = you were happy - lnl = you felt lonely - enj = you enjoyed life - sad = you felt sad - gng = you could not get going - Eft = you felt everything you did was an effort - slp = your sleep was restless |

**Figure s16.** The cross-lagged panel networks for first to second pre-COVID (wave 8: May 2016 – June 2017 🡪 wave 9: June 2018 – July 2019), second pre-COVID to first COVID (wave 9 🡪 COVID-19 wave 1: June – July 2020) and first COVID to second COVID (COVID-19 wave 1 🡪 COVID-19 wave 2: November – December 2020) time-points using the full-case dataset without imputation. The relationship of the CES-D-8 items is indicated by the arrow’s color (green = positive, red = negative) and the strength of the relationship is indicated by the arrow’s thickness (thicker = stronger). The placement of nodes is arbitrary and only for visualization purposes. Autoregressive effects are excluded from the graphical representation for a clearer visualization of the cross-lagged effects. The items were happy and enjoyed life were reverse coded.


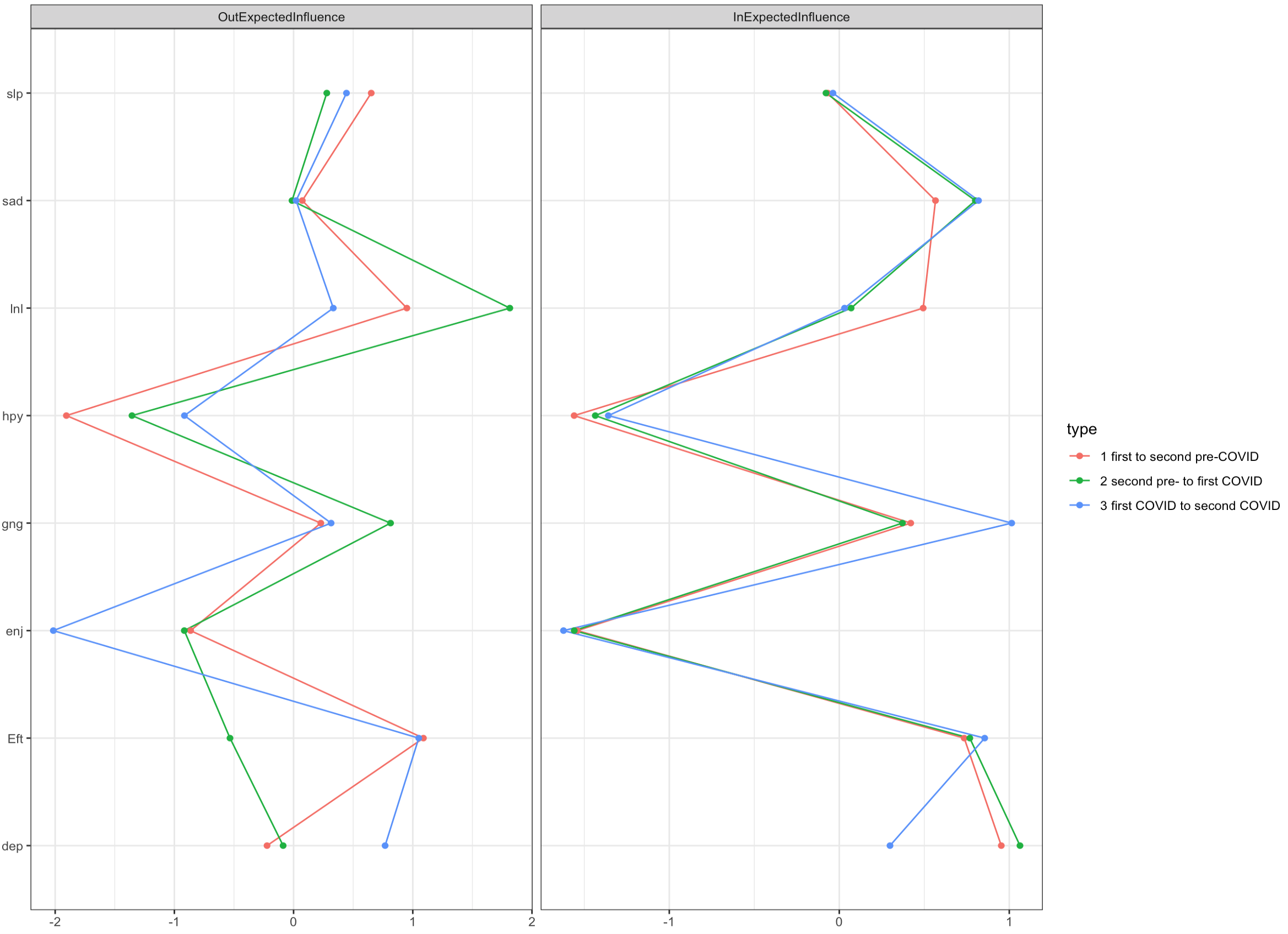


**Figure s17.** Symptom centrality estimates for the networks using z-values. Greater values indicate greater centrality. Out-expected-influence is the degree to which a symptom predicts other symptoms at the subsequent relevant point. In-expected-influence is the degree to which a symptom is predicted by other symptoms at the subsequent relevant point.

| **During the past week indicate whether…** | **Wave 8 (*N*= 7223)** | **Wave 9 (*N*= 7223)** | **COVID wave 1 (*N*= 7223)** | **COVID wave 2 (*N* = 7223)** |
| --- | --- | --- | --- | --- |
| Assessment year | 05/2016-06/2017 | 06/2018-07/2019 | 06/2020-07/2020 | 11/2020-12/2020 |
| Age (SD) | 67.87 (14.48) | 67.59 (14.07) | 70.07 (9.37) | 70.25 (9.19) |
| Female (%) | 4041 (55.95 %) | 4131 (56.67 %) | 3322 (57.03 %) | 3175 (56.76 %) |
| 1 …you felt depressed | *n* = 868 (12.02 %) | *n* = 793 (10.98 %) | *n* = 1309 (18.12 %) | *n* = 1438 (19.90 %) |
| 2 …you felt everything you did was an effort | *n* = 1436 (19.88 %) | *n* = 1464 (20.27 %) | *n* = 1925 (26.66 %) | *n* = 2041 (28.26 %) |
| 3 …your sleep was restless | *n* = 2639 (36.54 %) | *n* = 3112 (43.09 %) | *n* = 3309 (45.81 %) | *n* = 3470 (48.04 %) |
| 4 …you were happy | *n* = 6574 (91.02 %) | *n* = 6592 (91.26 %) | *n* = 6054 (83.82 %) | *n* = 5813 (80.48 %) |
| 5 …you felt lonely | *n* = 867 (12.00 %) | *n* = 821 (11.37 %) | *n* = 1388 (19.22 %) | *n* = 1421 (19.67 %) |
| 6 …you enjoyed life | *n* = 6605 (91.44 %) | *n* = 6583 (91.14 %) | *n* = 5804 (80.35 %) | *n* = 5590 (77.39 %) |
| 7 …you felt sad | *n* = 1405 (19.45 %) | *n* = 1314 (18.19 %) | *n* = 1798 (24.90 %) | *n* = 2218 (30.71 %) |
| 8 …you could not get going | *n* = 1422 (19.69 %) | *n* = 1451 (20.09 %) | *n* = 2120 (29.35 %) | *n* = 2371 (32.83 %) |
| Cronbach’s alpha, full scale (α) | 0.78 | 0.77 | 0.81 | 0.82 |
| Depressed affect (α) | 0.76 | 0.75 | 0.79 | 0.79 |
| Somatic complaints (α) | 0.45 | 0.44 | 0.50 | 0.52 |
| **Skewness/Kurtosis** | **Wave 8 (*N*= 7223)** | **Wave 9 (*N*= 7223)** | **COVID wave 1 (*N*= 7223)** | **COVID wave 2 (*N* = 7223)** |
| 1 …you felt depressed | 2.34/3.46 | 2.50/4.23 | 1.65/0.74 | 1.51/0.27 |
| 2 …you felt everything you did was an effort | 1.51/0.28 | 1.48/0.19 | 1.06/-0.89 | 0.97/-1.07 |
| 3 …your sleep was restless | 0.56/-1.69 | 0.28/-1.92 | 0.17/-1.97 | 0.08/-1.99 |
| 4 …you were happy | -2.87/6.23 | -2.92/6.54 | -1.84/1.37 | -1.54/0.36 |
| 5 …you felt lonely | 2.34/3.47 | 2.43/3.92 | 1.56/0.44 | 1.53/0.33 |
| 6 …you enjoyed life | -2.96/6.78 | -2.89/6.38 | -1.53/0.33 | -1.31/-0.29 |
| 7 …you felt sad | 1.54/0.38 | 1.65/0.72 | 1.16/-0.65 | 0.84/-1.30 |
| 8 …you could not get going | 1.52/0.32 | 1.49/0.23 | 0.91/-1.18 | 0.73/-1.47 |
| Omega total, full scale (ω_t_) | 0.84 | 0.83 | 0.86 | 0.87 |
| Depressed affect (ω_t_) | 0.81 | 0.80 | 0.83 | 0.83 |
| Somatic complaints (ω_t_) | 0.48 | 0.48 | 0.53 | 0.54 |

**Table 1.** Number and percentage of participants endorsing the dichotomous CES-D-8 items across waves, sum scores, internal consistencies, and item-level skewness and kurtosis for the imputed data. The items *were happy* and *enjoyed life* were reverse-coded and included in the model, leading to negative associations.

| **During the past week indicate whether…** | **Wave 8** | **Wave 9** | **COVID wave 1** | **COVID wave 2** |
| --- | --- | --- | --- | --- |
| Assessment year | 05/2016-06/2017 | 06/2018-07/2019 | 06/2020-07/2020 | 11/2020-12/2020 |
| 1 …you felt depressed | 324 (4.49 %) | 1347 (18.65 %) | 2232 (30.90 %) | 2424 (33.56 %) |
| 2 …you felt everything you did was an effort | 324 (4.49 %) | 1347 (18.65 %) | 2232 (30.90 %) | 2425 (33.57 %) |
| 3 …your sleep was restless | 322 (4.46 %) | 1346 (18.63 %) | 2236 (30.96 %) | 2428 (33.61 %) |
| 4 …you were happy | 334 (4.62 %) | 1361 (18.84 %) | 2257 (31.25 %) | 2444 (33.84 %) |
| 5 …you felt lonely | 322 (4.46 %) | 1348 (18.66 %) | 2236 (30.96 %) | 2431 (33.66 %) |
| 6 …you enjoyed life | 329 (4.55 %) | 1360 (18.83 %) | 2263 (31.33 %) | 2449 (33.91 %) |
| 7 …you felt sad | 323 (4.47 %) | 1350 (18.69 %) | 6074 (84.09 %) | 2435 (33.71 %) |
| 8 …you could not get going | 324 (4.49 %) | 1351 (18.70 %) | 2249 (31.14 %) | 2434 (33.70 %) |

**Table 2.** Table showing the missing values in the CES-D-8 items for each item and each wave, total and in percent of the dataset with *N*= 7,223 participants.

| **First to second pre-COVID**  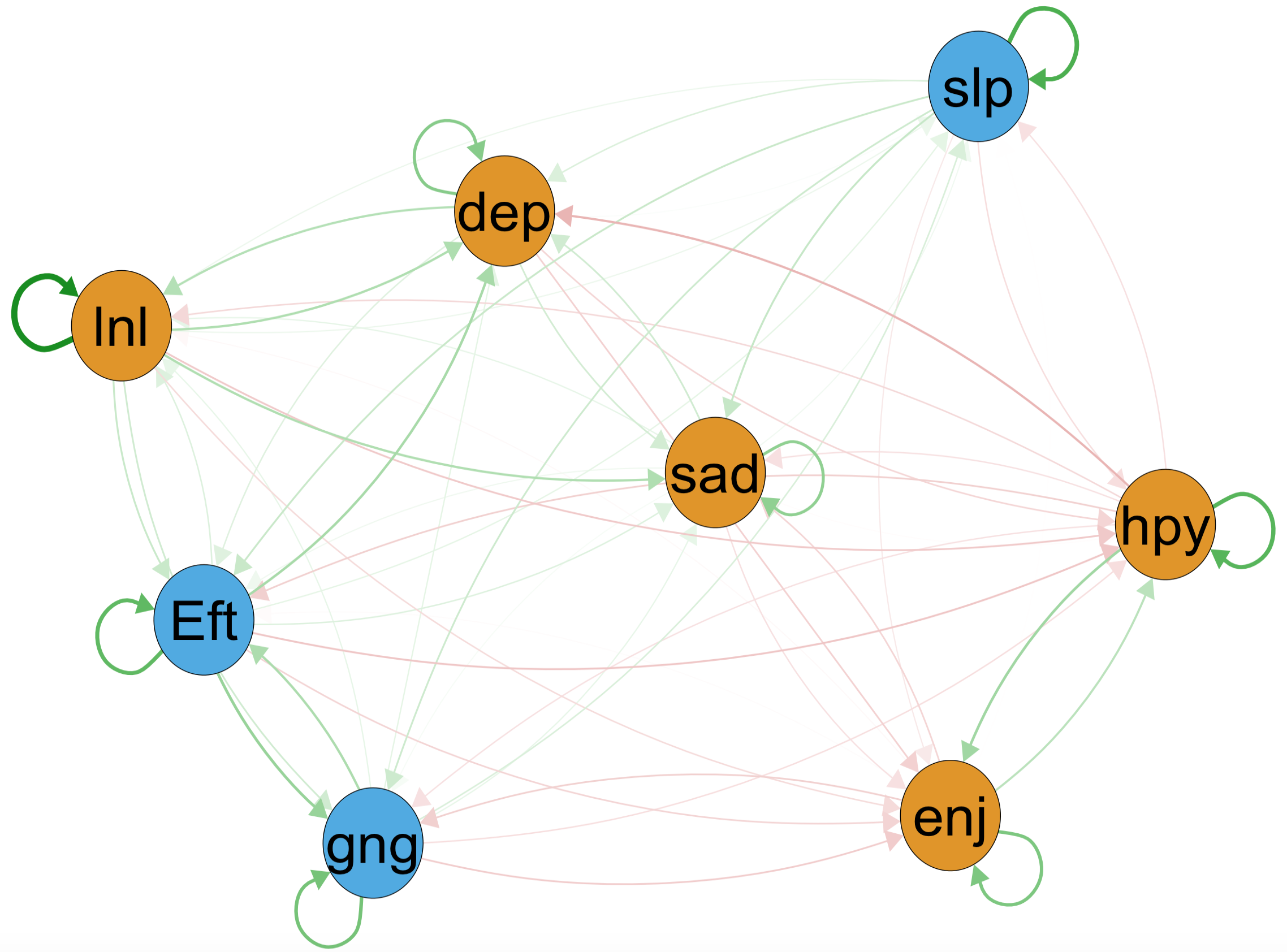 | **Second pre-COVID to first COVID**  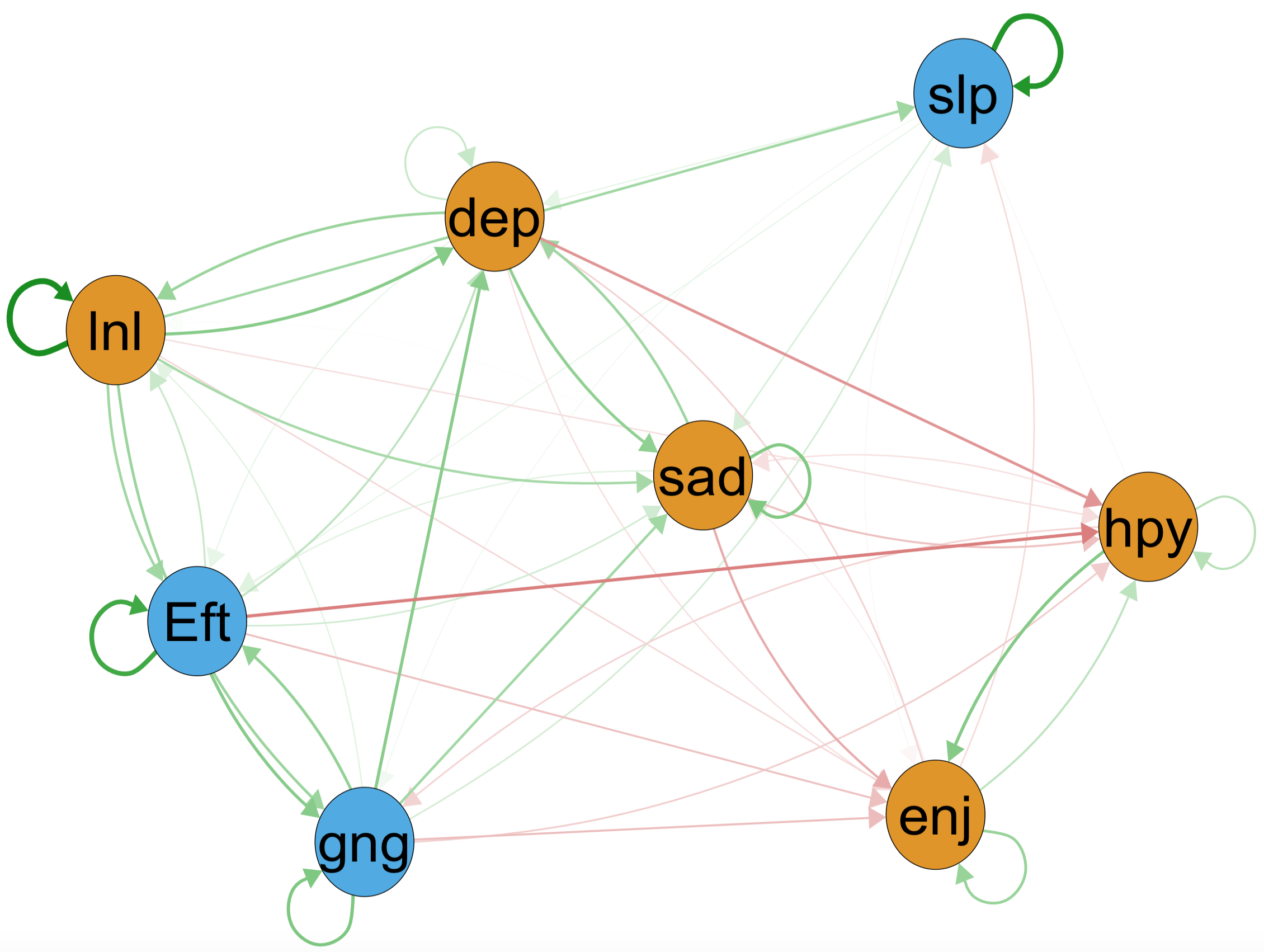 |
| --- | --- |
| **First COVID to second COVID**  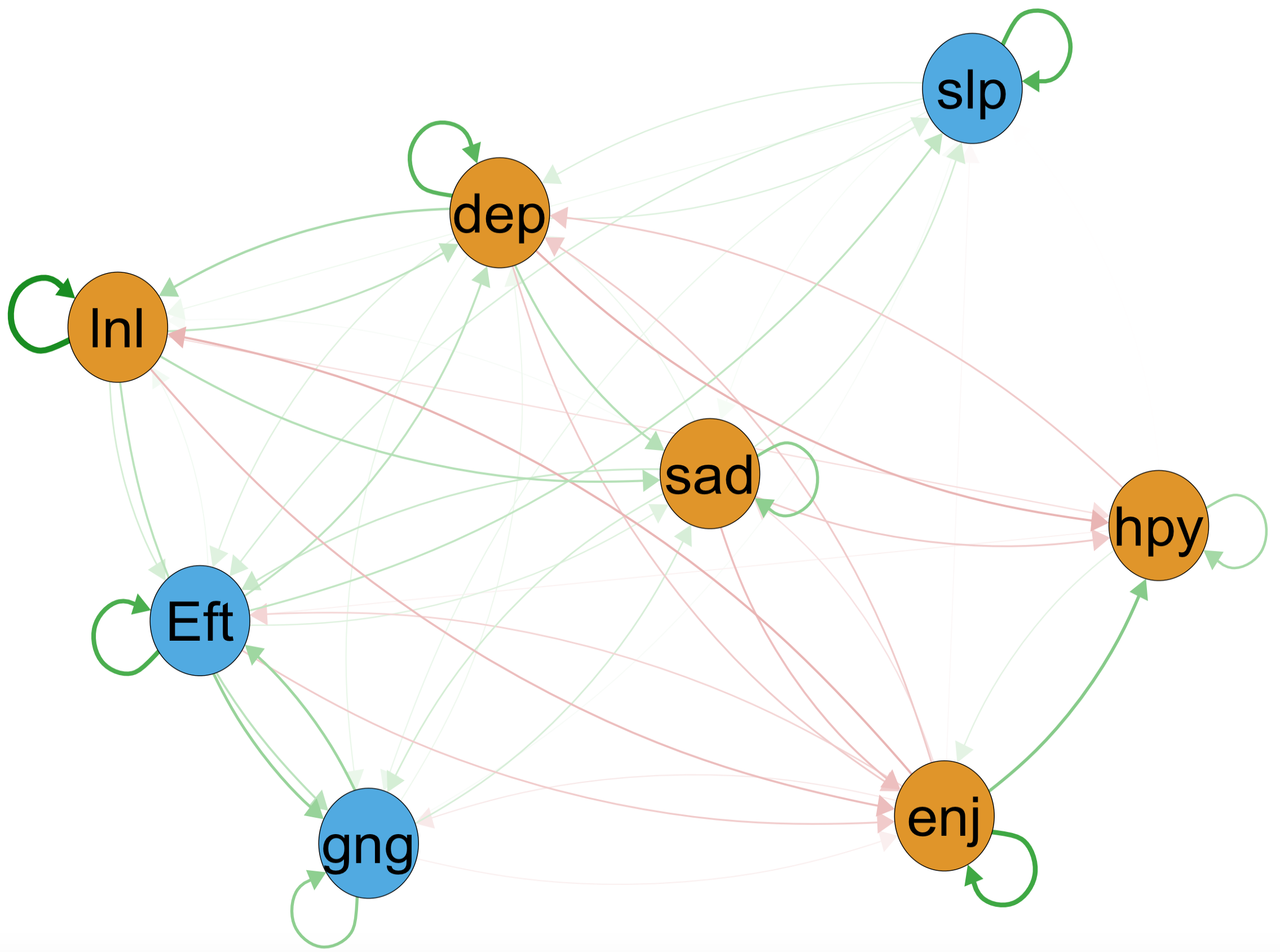 | - dep = you felt depressed - hpy = you were happy - lnl = you felt lonely - enj = you enjoyed life - sad = you felt sad - gng = you could not get going - Eft = you felt everything you did was an effort - slp = your sleep was restless |

**Figure s18.** The cross-lagged panel networks for first to second pre-COVID (wave 8: May 2016 – June 2017 🡪 wave 9: June 2018 – July 2019), second pre-COVID to first COVID (wave 9 🡪 COVID-19 wave 1: June – July 2020) and first COVID to second COVID (COVID-19 wave 1 🡪 COVID-19 wave 2: November – December 2020) time-points using the dataset with imputation. The relationship of the CES-D-8 items is indicated by the arrow’s color (green = positive, red = negative) and the strength of the relationship is indicated by the arrow’s thickness (thicker = stronger). The placement of nodes is arbitrary and only for visualization purposes. Autoregressive effects are included into the graphical representation. The items were happy and enjoyed life were reverse coded.

| **First to second pre-COVID**  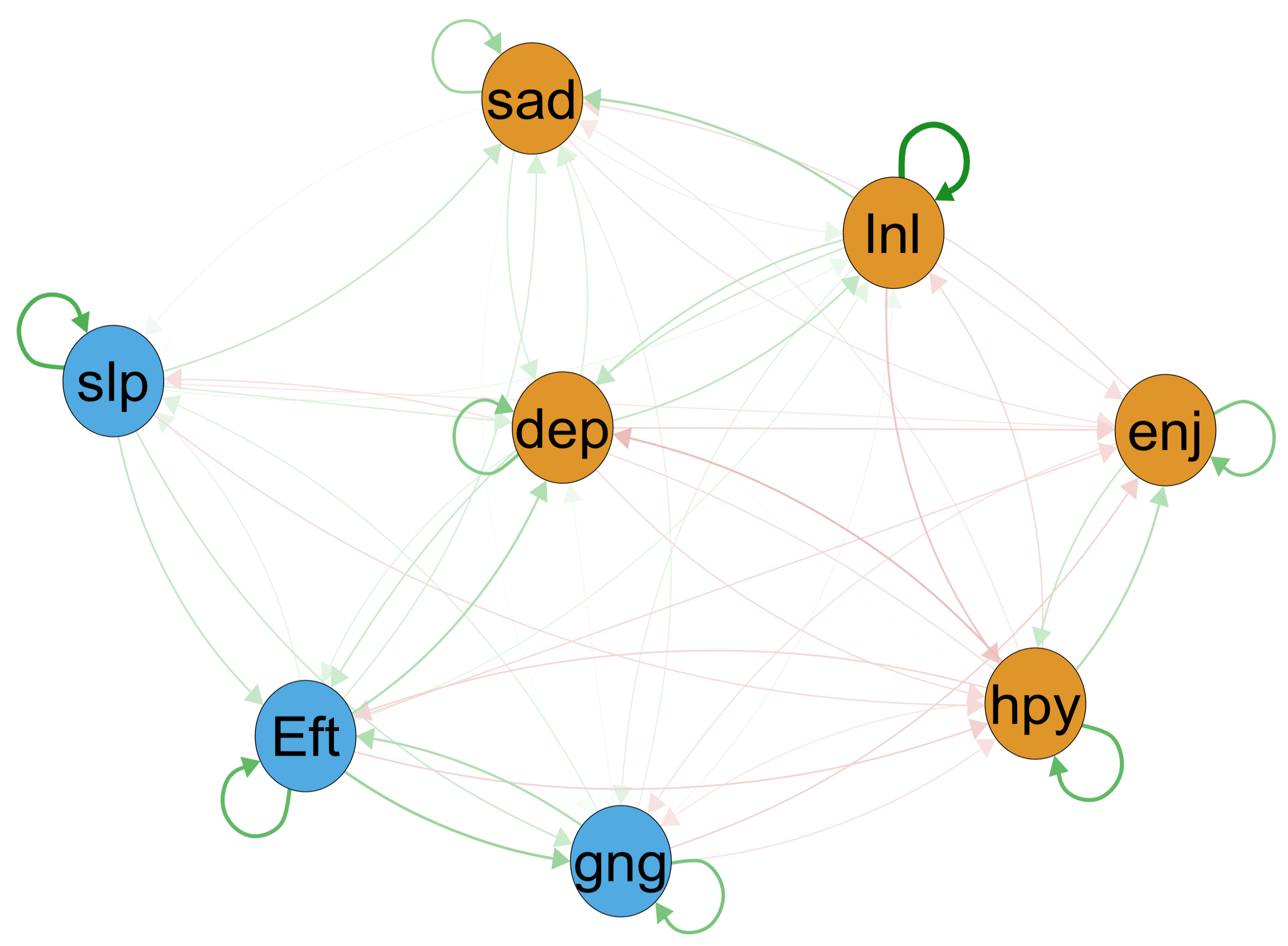 | **Second pre-COVID to first COVID**  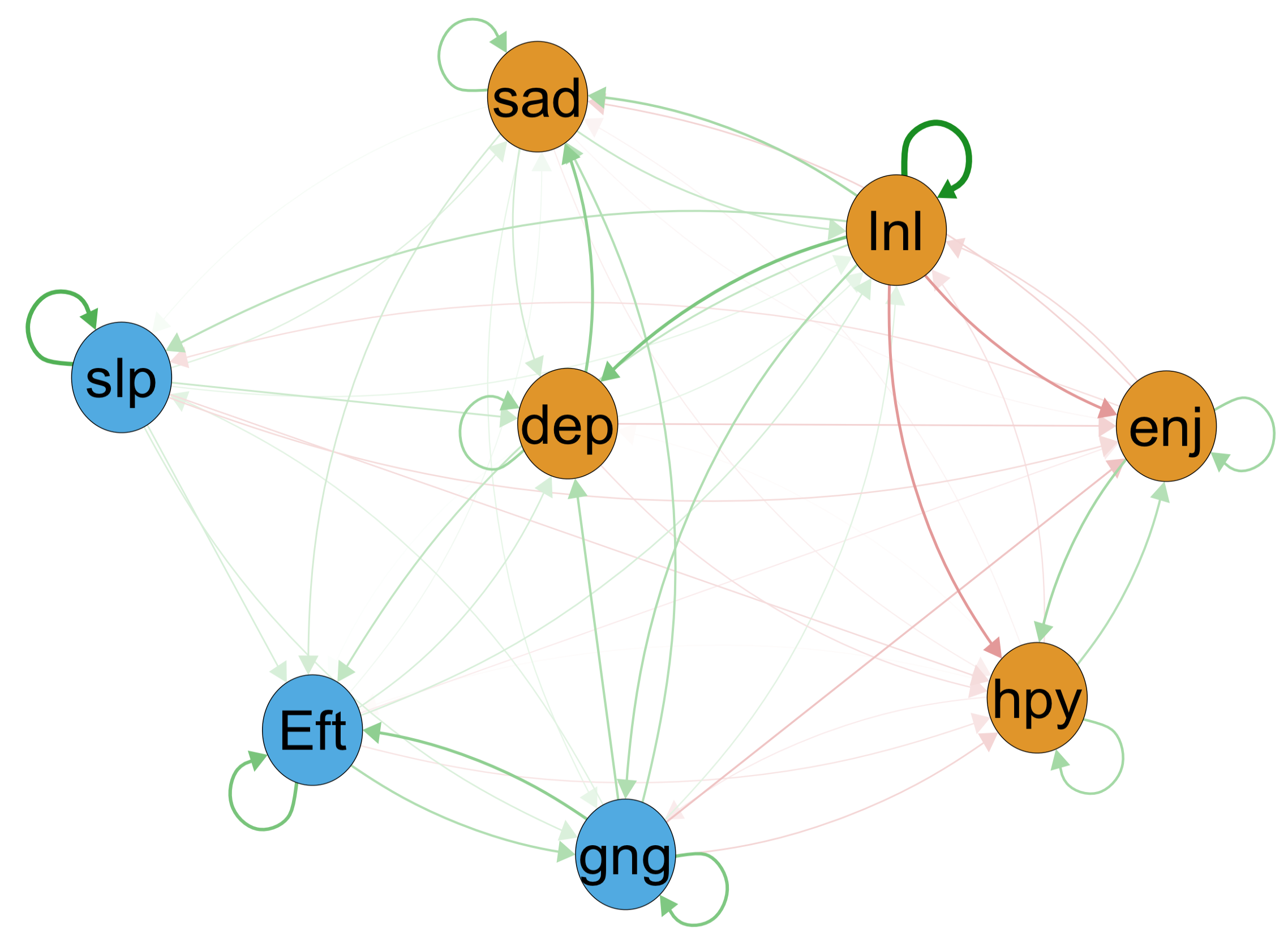 |
| --- | --- |
| **First COVID to second COVID**  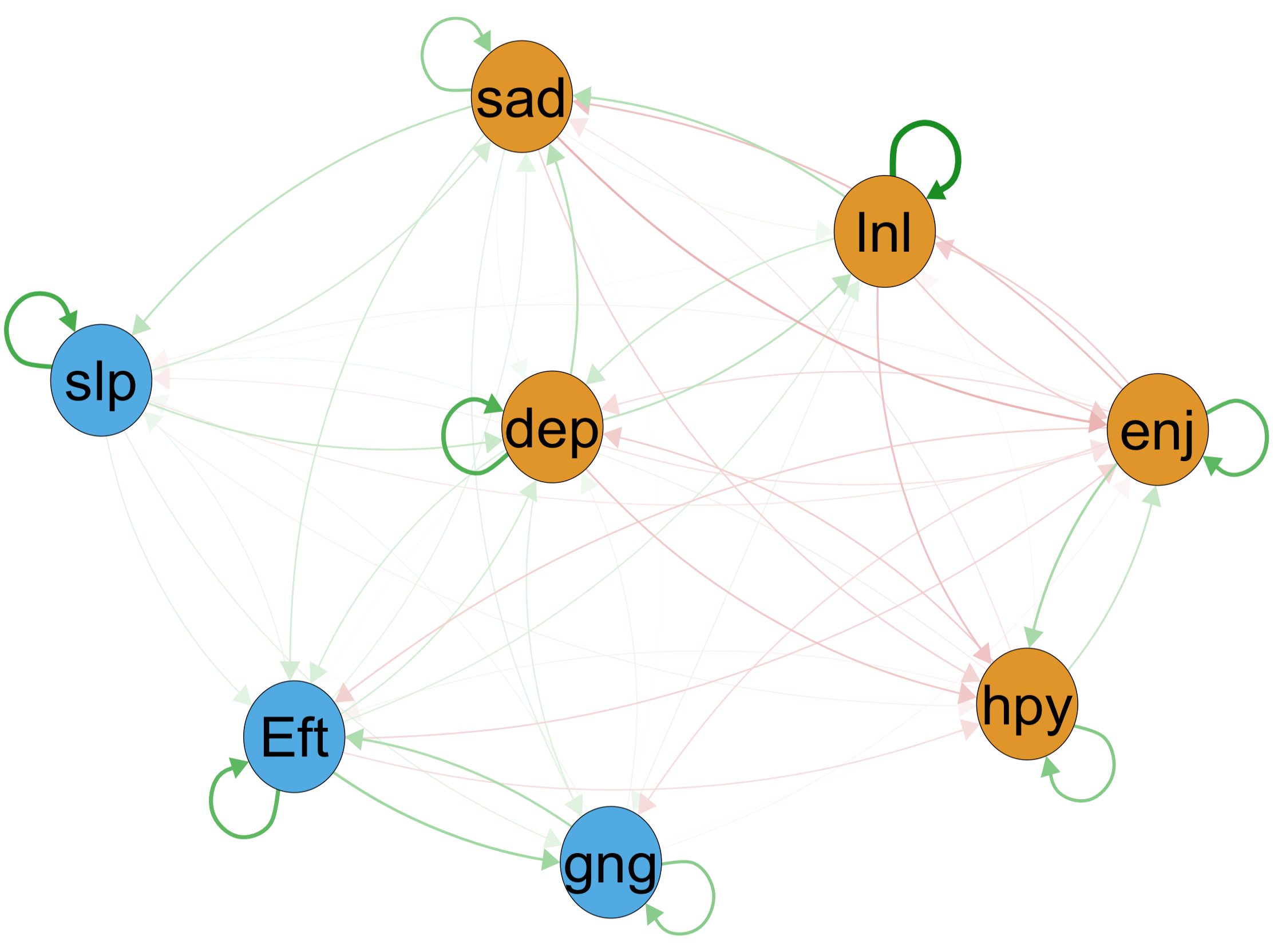 | - dep = you felt depressed - hpy = you were happy - lnl = you felt lonely - enj = you enjoyed life - sad = you felt sad - gng = you could not get going - Eft = you felt everything you did was an effort - slp = your sleep was restless |

**Figure s19.** The cross-lagged panel networks for first to second pre-COVID (wave 8: May 2016 – June 2017 🡪 wave 9: June 2018 – July 2019), second pre-COVID to first COVID (wave 9 🡪 COVID-19 wave 1: June – July 2020) and first COVID to second COVID (COVID-19 wave 1 🡪 COVID-19 wave 2: November – December 2020) time-points using the dataset without imputation. The relationship of the CES-D-8 items is indicated by the arrow’s color (green = positive, red = negative) and the strength of the relationship is indicated by the arrow’s thickness (thicker = stronger). The placement of nodes is arbitrary and only for visualization purposes. Autoregressive effects are included into the graphical representation. The items were happy and enjoyed life were reverse coded.

**Figure s20.** COVID-19 stringency index of the UK regarding the relevant periods June – July 2020 and November – December 2020. Data from https://ourworldindata.org/coronavirus#explore-the-global-situation


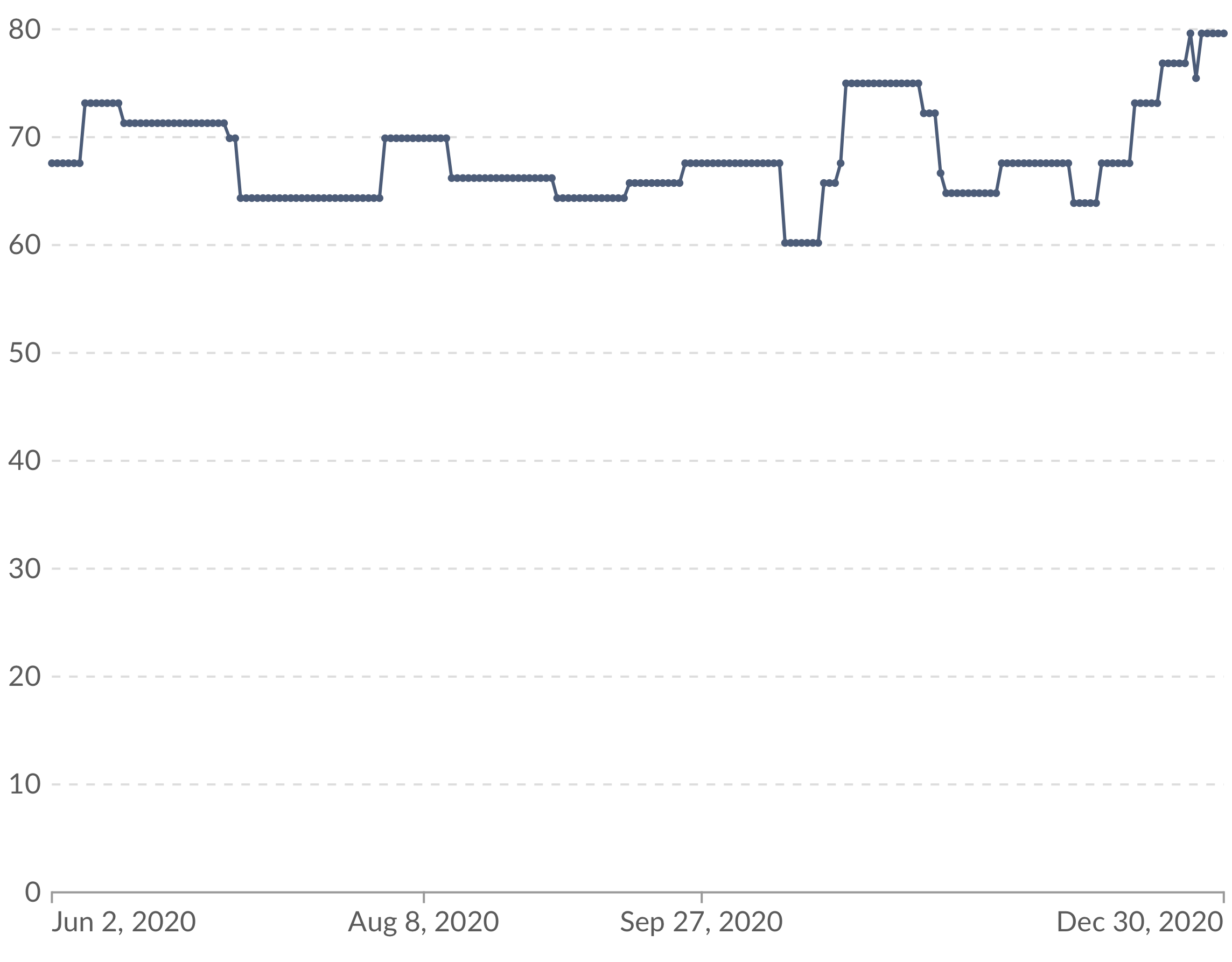

Supplement: Supplementary file 1 — Supplementary material [file mmc1.docx]
